# Individual Differences in Strategy and the Item-Position Effect in Reasoning Ability Measures

**DOI:** 10.3390/jintelligence13070077

**Published:** 2025-06-26

**Authors:** Helene M. von Gugelberg, Stefan J. Troche

**Affiliations:** Department of Psychology, University of Bern, 3012 Bern, Switzerland; stefan.troche@unibe.ch

**Keywords:** Raven’s Advanced Progressive Matrices, strategy, item-position effect, eye tracking, rule learning

## Abstract

Despite the high similarity of reasoning ability items, research indicates that individuals apply different strategies when solving them. The two distinct strategies are response elimination and constructive matching. The latter, frequently showing a positive correlation with reasoning ability, entails the individual systematically investigating the presented problem matrix of an item before scanning the response alternatives. To further understand the sources of individual differences in strategy use during test taking, three different eye-tracking metrics were investigated in participants (*N* = 210) solving the Raven’s Advanced Progressive Matrices (APM). Relying on the fixed-links modeling approach, bifactor models were fit to the data. The latent model approach revealed, in line with other research, a positive correlation between reasoning ability and constructive matching. The results further indicated that a change in strategy use was correlated with the item-position effect and not reasoning ability. The former exhibited a different direction of effect, depending on the eye-tracking metric analyzed. When investigating the toggle rate, the participants used more constructive matching towards the end of the APM. The proportional time to first fixation on response alternatives indicated less constructive matching as the test progressed, and the proportional time on the problem matrix exhibited no distinct pattern regarding a change in strategy use. These diverging results point towards the possibility of a more nuanced problem-solving behavior than previously assumed. By including the item-position effect in the analyses, the increasing individuals differences in problem-solving behavior can be taken into account, which could be a necessary step in attaining a more comprehensive understanding of problem-solving behavior.

## 1. Introduction

**Figure 1:**
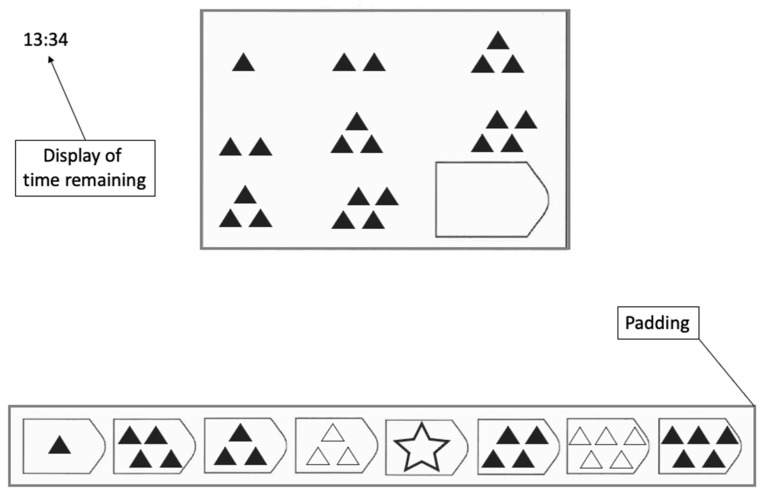
Fictitious example item of the APM.

Raven’s Advanced Progressive Matrices (Raven et al. 1998) have been developed to measure reasoning ability and, thereby, a core aspect of general intelligence (Raven 2000; Spearman 1927). Each APM item is composed of a 3 × 3 matrix with figural stimuli in eight of the nine entries, while the bottom right entry is missing (see Figure 1 for an illustrative example). The participant’s task is to identify the rule(s) connecting the stimuli within each row or column to infer what the missing entry should look like and to select the correct option out of eight response alternatives. Although the relationship between APM test scores and general intelligence seems to be weaker than commonly assumed (Gignac 2015), empirical findings support the substantial relationship between APM test scores and general intelligence (e.g., Marshalek et al. 1983). This led to the frequent administration of the APM in research when general intelligence was assessed by a single test instead of a comprehensive test battery. As a result, many insights into the correlates of intelligence (for example, cognitive and neural) are based on the APM.

Another line of research focused on the cognitive processes required by the APM items and their underlying rules (Carpenter et al. 1990; Lozano and Revuelta 2020; Verguts and De Boeck 2002) and revealed individual differences in problem-solving strategies applied to the items (e.g., Gonthier and Thomassin 2015; Hayes et al. 2015; Loesche et al. 2015; Snow 1978; Vigneau et al. 2006). Studies on problem-solving strategies in aptitude tests with a multiple choice format identified two distinct test-taking strategies (and sometimes a third strategy, likely a mix of the two distinct strategies—see Jarosz et al. 2019; Liu et al. 2023) through verbal protocols and the observation of eye movements, namely the constructive matching and response elimination strategies (Bethell-Fox et al. 1984; Snow 1978).

When individuals apply the constructive matching strategy, they spend much of the time on the matrix, identifying the underlying rules, constructing the missing entry, to then selecting the matching entry from the response alternatives. With the response elimination strategy, individuals eliminate step-by-step, non-viable solutions from the response alternatives until the correct solution is found. These strategies were investigated and identified in the APM by means of eye-tracking data (Vigneau et al. 2006), verbal protocols (Jarosz et al. 2019), and questionnaires (Gonthier and Thomassin 2015). Independent of how the strategy was assessed, constructive matching was associated with higher test scores compared to response elimination (e.g., Gonthier and Roulin 2020; Jastrzębski et al. 2018).

Eye tracking is an objective instrument used to assess strategy use, and test takers are not asked about their problem-solving behavior while completing the test. Self-report questions or think-aloud protocols after each item can sometimes lead to a change in problem-solving behavior (i.e., reactivity). Despite an abundance of information on eye-tracking metrics offered on every item for every individual (Vigneau et al. 2006; Hayes et al. 2015; Laurence et al. 2018), they do not provide insight into other possible cognitive processes (e.g., verbal rehearsals). Hence, eye tracking provides only information about strategy use that translates directly to eye movements. Three of the frequently used metrics are the toggle rate, proportional time on the matrix, and latency to the first fixation on response alternatives (Laurence and Macedo 2023).

To quantify the toggle rate, the fixations an individual makes while solving an item are assessed and divided by item latency. A toggle occurs when an individual first shows a fixation on the matrix, followed by a fixation on the response alternatives, or vice versa. Constructive matching leads to few toggles since most of the time is spent on the problem matrix to analyze the rules and to mentally construct a solution. Using response elimination, however, necessitates frequently alternating between the matrix and the response alternatives to check whether a response alternative fits the empty entry in the matrix. This results in a high number of toggles. Thus, constructive matching and response elimination differ in the frequency of toggles made while solving the item. Since more time spent on an item provides more opportunities to toggle, the absolute number of toggles is divided by the item’s latency.

The proportional time spent inspecting the problem matrix is another frequently used metric for strategy use within eye tracking (e.g., Li et al. 2022). It is calculated using the time a participant spends inspecting the problem matrix of an item divided by the total amount of time a participant spends on the said item. Since the constructive matching strategy includes a systematic analysis of the problem matrix to construct a possible answer, this strategy involves much time spent on the problem matrix in relation to the total time spent on an item. Hence, a larger value in this metric is indicative of constructive matching. In comparison, a smaller value indicates that participants spent proportionally more time looking at response alternatives, which is typical for response elimination.

Latency to first fixation on response alternatives can also be used to distinguish between the two strategies (e.g., Vigneau et al. 2006). When a participant spends only a small amount of time on the problem matrix before switching to the response alternatives, it is seen as an indicator of response elimination. On the contrary, higher latency to first fixation on response alternatives indicates constructive matching since more time passes before the participant inspects the possible response alternatives.

Why different strategies are used and why they lead to different results is an ongoing debate. However, there is some evidence that test demands (e.g., Raden and Jarosz 2022) and available mental capacities (Gonthier and Thomassin 2015; Jarosz et al. 2019; Li et al. 2022) influence what strategy an individual applies to an item. Summarized, strategy use is influenced by the interrelationship of the mental resources of an individual and perceived item properties. Additionally, Bethell-Fox et al. (1984) and Snow (1980) found that individuals switched from constructive matching to response elimination as a fallback strategy when their capacity to hold rules and manipulate objects in their minds was exceeded.

Hence, according to Bethell-Fox et al. (1984) and Snow (1980), a shift towards more response elimination should be observed in a test with increasing item difficulty as the likelihood increases that item demands exceed the test takers’ capacity. Gonthier and Roulin (2020) reported such a shift in test-taking strategies in the APM based on an analysis of self-report questionnaires. Their participants progressively engaged in more response elimination as the items became more difficult. This shift in strategy was mediated by working memory capacity, as individuals with a higher working memory capacity used constructive matching for more items as the test progressed compared to individuals with a lower working memory capacity. This is in line with the assumptions made by Bethell-Fox et al. (1984) and Snow (1980) since individuals with a higher working memory capacity should be better able to handle increasing item difficulty (e.g., the simultaneous processing of several rules). Hence, they can use constructive matching on more items and more difficult items.

Jarosz et al. (2019) also observed a shift towards less constructive matching with increasing item difficulty. As the test progressed, individuals used less and less constructive matching. Further analyses revealed that both working memory capacity and item difficulty influenced the likelihood of using constructive matching from item to item, although their interaction was not significant. Another noteworthy observation by Jarosz et al. (2019) was that individuals with a high working memory capacity were more likely to attempt the first APM item using constructive matching compared to individuals with a low working memory capacity. These results suggest that, with increasing item difficulty, strategy use generally shifts towards less constructive matching. Simultaneously, a high working memory capacity enables more constructive matching.

Furthermore, Perret and Dauvier (2018) found, in a sample of children, that the ability to adapt one’s response latency was specifically linked to performance. In their reasoning ability test, performance increased not only as the children grew older but especially for those children who showed the largest adaptation in response times between easy and difficult items. In a test with increasing item difficulty, such as the APM, this would translate to progressively longer response times as the test progresses, especially for participants with higher reasoning abilities. Along this line, Gonthier et al. (2024) found that older children used more constructive matching on difficult items compared to younger children. Hence, a higher ability came with better adaptability regarding strategy use for difficult items. This would mean a shift towards more constructive matching within the APM since item difficulty increases throughout the test.

Summarized, there is evidence for adaptability or change in test-taking strategies, yet there is not enough evidence to understand what impacts strategy use and the possible changes occurring therein, especially results regarding the direction of the shift in strategy use diverge (an increase in response elimination (Snow 1980) or an increase in constructive matching (Gonthier et al. 2024)).

Regardless of the direction, such changes in strategy use during a test likely lead to a systematic increase in variance regarding the response behavior from item to item. This corresponds to a well-replicated finding in structural investigations of the APM, namely, the item-position effect (Lozano 2015; Ren et al. 2014, 2017b; Schweizer et al. 2009, 2021; Zeller et al. 2017). An item-position effect in reasoning tests can be assumed when the quality of a response to an item is not only influenced by the test takers’ reasoning ability but also by the position at which the item is presented. Studies found that data description improved when, in addition to reasoning ability, a second latent variable was introduced to a measurement model (Ren et al. 2014, 2017b; Schweizer et al. 2009, 2021), specifically when this second latent variable had its factor loadings monotonically increase (linearly or quadratically) from item to item. This indicates that, in addition to reasoning ability, a second source explained a significant amount of variance in response behavior, with its relevance increasing from item to item and, therefore, is referred to as the item-position effect.

Studies on the meaning of the item-position effect in APM responses revealed that item difficulty was not sufficient to explain the item-position effect since it was also observed when the items were presented in quasi-random order (Zeller et al. 2017; Schweizer et al. 2021) or when item difficulty was held constant in simulated data (Schweizer and Troche 2018). Other studies showed that the item-position effect was related to impulsivity (Lozano 2015, but also see Ren et al. 2017b), working memory updating, and shifting (Ren et al. 2017a), as well as to proactive control (von Gugelberg et al. 2021) and rule learning (Ren et al. 2014; Schweizer et al. 2021; von Gugelberg et al. 2025). To the best of our knowledge, the item-position effect has not been investigated as to whether it is related to a change in the test-taking strategy or not.

### Current Study

The main objective of the present study was to examine whether there is a systematic relationship between the item-position effect and a shift in strategy use. To investigate this objective, a number of preconditions must be met. In particular, an item-position effect must be detectable in the data, and eye-tracking metrics must reveal a shift in strategy use.

Hence, the first analysis investigated the presence of an item-position effect in the APM responses. For this purpose, different models were fit to the APM responses to discern whether the better-fitting model included an item-position effect. Due to the consistent finding of an item-position effect in the APM items (e.g., Schweizer et al. 2012; Zeller et al. 2017), we expected to obtain a better data description when a latent item-position variable was added to a latent reasoning variable in a bifactor model.

This analysis was followed by investigating individual differences in the applied test-taking strategy. More specifically, we investigated whether individual differences in the toggle rate, proportional time on matrix, and latency to first fixation on response alternatives were constant across the APM items (one-factor model) or showed a systematic shift (bifactor model). For this purpose, the bifactor model consisted of a basic latent variable (with estimated factor loadings for the eye-tracking metric) and a latent variable with factor loadings fixed to a linear or quadratic increase, from the first to the last item. This statistical approach allowed for the representation of basic individual differences in the strategy applied (as in Vigneau et al. 2006) but also for a shift in strategy from the first to the last item (as in Gonthier and Roulin 2020).

Regarding the toggle rate, the second latent variable describes that individuals increasingly differ in the number of toggles made in relation to item latency as the test progresses. In other words, the latent variable depicting the change in toggle rate would indicate to what extent participants differ in adapting their toggle rate as the test progresses.

Similarly, in the model fit to the proportional time spent on the problem matrix, the second latent variable depicts the change in proportional time spent on the problem matrix. This variable informs about whether the time participants allocate to inspect the problem matrix grows increasingly differently between participants. If, for the proportional time to the first fixation on the response alternatives, a bifactor model describes the data well, the latent variable with increasing factor loadings depicts an increasing difference between participants as to when they first look at the response alternatives in the given item.

The main objective was then to examine the interplay among the latent variables extracted from the APM responses and the eye-tracking metrics. We expected that the general tendency to use constructive matching (rather than response elimination) was related to reasoning ability, as previously reported (e.g., Jarosz et al. 2019). We also expected that if a strategy shift in the eye-tracking metrics could be identified, this shift would be related to the item-position effect in APM responses.

## 2. Methods

### 2.1. Participants

Participants were recruited through the university, other local institutions, online, or directly by the contributors of this project. Psychology undergraduates received course credit, and participants without a university entrance qualification received 20 CHF for participating. A total of 217 individuals participated. One participant did not declare their gender, age, or highest level of education. The remainder of the sample had a mean age of 27.5 years (SD = 11.9 years). A total of 136 participants described themselves as female, 79 as male, and 1 person chose “other”. A total of 136 participants reported having a university entrance qualification as their highest level of education, 53 had a bachelor’s degree or higher, and 27 participants had neither. All participants reported normal or corrected-to-normal vision and gave written informed consent. The study’s protocol was approved by the local ethics committee of the Faculty of Human Sciences of the University of Bern (No. 2020-07-00001).

### 2.2. Raven’s Advanced Progressive Matrices

The second set of the APM, with its 36 items (Raven et al. 1998), was presented via Psychopy v2020.1.2 software (Peirce et al. 2019) on a computer monitor (a Dell 18-inch monitor with a resolution of 1280 × 1024 pixels) with a width of 34 cm and a height of 27 cm. The participants were seated in front of the monitor at a distance of 85 cm, ensured by a chin–forehead rest. The black and white stimuli of an APM item consisted of a 3 × 3 problem matrix with eight geometrical figures and the bottom right entry missing (see Figure 1). Underneath the problem matrix, eight response alternatives were presented. The problem matrix was displayed with a width of 14.9 cm and a height of 10.8 cm. The padding of response alternatives had a width of 30 cm and a height of 2.5 cm. The participants were asked to choose a response alternative by clicking on it with a wired computer mouse.

After being instructed in accordance with the test manual, the participants completed two example items before the test started properly. Contrary to the manual, a time limit was set at 30 min for the completion of the 36 items. The response to each APM item was coded with “1” for a correct or “0” for an incorrect response.

### 2.3. Eye Tracking Metrics

With the EyeLink 1000 Plus system (SR Research 2016) (SR Research EyeLink: Ottawa, ON, Canada), eye movements were tracked by an infrared video camera with a 500 Hz sampling rate. For the analysis of eye movement data, monocular eye data were used, whereby the eye with the smaller measurement error was automatically selected after the calibration and validation procedure. The fixation duration threshold was set at 100 ms (e.g., Martarelli and Mast 2013). Areas of interest were created for the problem matrix and the response alternatives. The area of interest for the response alternatives had additional padding (Bojko 2013). No padding was added to the interest area of the problem matrix since it already contained blank space around the matrix entries (see Figure 1).

Data of each individual and every item were visually inspected for potential drift with the EyeLink Data viewer (SR Research EyeLink: Ottawa, ON, Canada). Drift occurs when a participant moves their head after the calibration and validation processes. This leads to a systematic drift in a certain direction, leaving many or even all fixations on blank areas of the screen. Whenever such a systematic drift was detected, all fixations were moved in cohesion to have them located in reasonable areas of the item.

To calculate the proportional time spent on the problem matrix, the duration of all fixations on the problem matrix was summed up and then divided by item latency. The first fixation on any given interest area can be directly exported from the EyeLink Data viewer. Hence, no additional calculations had to be made to create the variable for the first fixation on response alternatives.

To create the toggle rate, a fixation report for each participant was created, and the number of toggles was calculated. A toggle was defined as a fixation on the matrix followed by a fixation on the response alternatives or vice versa. All fixations outside of the defined interest areas were recoded with the value of the interest area of the previous fixation. Therefore, if a participant first fixated on the matrix, then stared at empty white space outside of the interest areas (possibly thinking), and then showed the next fixation on the response alternatives, this was counted as a toggle. Without the recoding, this type of scan path would not have been counted as a toggle, although it qualified as an alternation between matrix and response alternatives during the solving process. For each item, the number of toggles was divided by the time spent on the corresponding item (item latency), resulting in the toggle rate (as in Laurence et al. 2018; Gonthier et al. 2024; Vigneau et al. 2006).

### 2.4. Procedure

Data were collected as part of a larger two-part study. During the first session, the participants completed several tasks not relevant to the present study and answered a socio-demographic questionnaire. The second session took place at least 24 h after the first session. During the second session, eye movement data and other physiological data were collected during the completion of several tasks. After attaching all electrodes and instructing the participants about the chin–forehead rest, the data collection of the second session started with the participants completing the APM.

The commencement of the APM initialized the Eyelink 1000 Plus system (SR Research 2016), prompting the experimenter to calibrate the eye tracker. After successful calibration, a validation was completed. A standard 9-point calibration and validation procedure was applied until an eye-tracking error below 0.8° was obtained. After reading the instructions of the APM and solving two example items, participants had 30 min to solve the APM items. The remaining time was displayed in the top left corner of the screen. Each item was separated by a time interval of 2 s. During this interval, a fixation cross was presented in the middle of the screen. When the time limit was exceeded or all 36 items were answered, the participants completed other tasks not relevant to the present study.

### 2.5. Statistical Analysis

Statistical analyses were conducted in R (v4.5.0) with packages lavaan (v0.6.17, Rosseel 2012) and psych (v2.5.3, Revelle 2015). All confirmatory factor analyses were run with robust maximum likelihood estimation.

**Figure 2:**
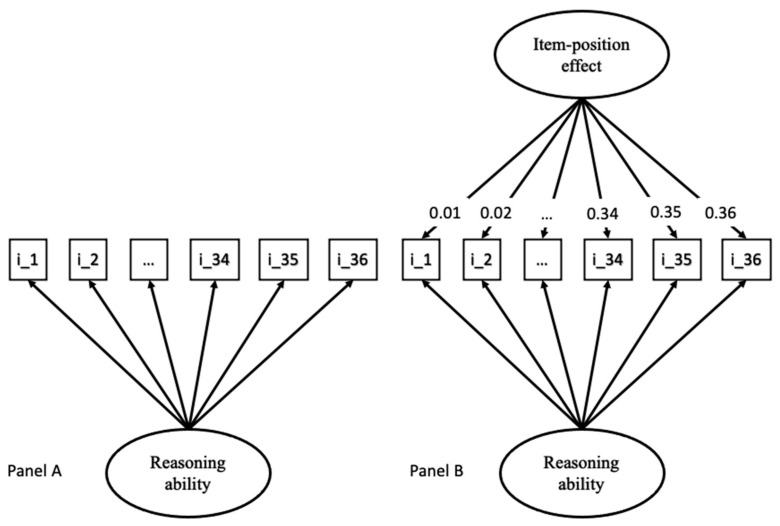
Illustration of one-factor and bifactor models for APM responses. **Panel A** illustrates a one-factor model with one factor for reasoning ability. **Panel B** shows a bifactor model with an item-position effect linearly increasing from item to item.

For the first objective, we analyzed whether an item-position effect emerged in the APM item responses. In line with prior research on the item-position effect, we fit three different models to the APM responses (e.g., Ren et al. 2014). The goal was to analyze whether a one-factor model (Figure 2, **Panel A**) or a bifactor model (Figure 2, **Panel B**) better described the variance and covariance of the data. For the bifactor models, a second latent variable was introduced. This second latent variable had its factor loadings set to a linear or quadratic increase from the first to the last item (Schweizer and Troche 2018). These factor loadings are not set arbitrarily but are based on theoretical assumptions on the possible factors underlying the second latent variable. This second latent variable depicts an increasing change in individual differences throughout test taking. The correlation between the two latent variables of the bifactor model was set to zero (cf., Markon 2019). The decision between the two models was based on the evaluation of the model fit (see below) and variance parameters, which should be statistically significant for both latent variables in order to argue that they explain a meaningful portion of the variance in the data (e.g., Lozano 2015; Ren et al. 2017b).

Due to the binary nature of the APM response data, probability-based covariances were used as input for the APM response models to allow for a threshold-free approach (Schweizer 2013). Factor loadings were additionally weighed by the item’s standard deviation (*SD*) to account for the difference between binary data distribution and normal distribution (Schweizer et al. 2015). This was done by setting the starting value of the factor loading estimation equal to the respective item’s standard deviation (this works similarly to pre-multiplication; see Rosseel 2012).

For the second objective of this study (investigating individual differences in the applied test-taking strategy), the same three models were fit to the data for each eye-tracking metric, albeit without additional weighting by the item’s standard deviation of factor loadings since the data were not binary.

The third objective of the current study was to examine the interplay among the latent variables extracted from the APM responses and the selected eye-tracking metrics. Hence, three full models, with the model exhibiting a superior data description for the APM responses and each of the respective eye-tracking metrics, were estimated.

The measurement models were evaluated by the following goodness-of-fit indices and criteria (DiStefano 2016): Comparative Fit Index (CFI; values above 0.90 for an acceptable fit, and values above 0.95 for a good fit), Root Mean Squared Approximation (RMSEA; values below 0.08 for an acceptable fit, and below 0.06 for a good fit), and Standardized Root Mean Square Residual (SRMR; values below 0.10 for an acceptable fit and below 0.08 for a good fit).

Models were compared with the Akaike Information Criterion (AIC). The AIC adjusts for parsimony and penalizes model complexity. Therefore, the AIC is an important criterion when comparing one-factor and bifactor models, where a lower AIC indicates a better model fit (DiStefano 2016). Further, higher values of at least 0.010 in the CFI indicate a better fit (Chen 2007). Data and R-script with the exact factor loadings for the analysis are available in the Supplementary Materials.

## 3. Results

Seven participants had faulty eye-tracking data that were caused by a recording failure. Hence, only 210 participants had complete data. With the strict time limit implemented in this study, not all participants managed to complete all 36 items. Only 164 participants completed all items, indicating that the results toward the end were clearly influenced by the implemented time limit. However, all 210 participants completed the first 22 items.

To only include 22 items in the analysis would increase the risk of not detecting a shift in strategy, as observed by Gonthier and Roulin (2020), or the item-position effect, and only including participants who completed all 36 items comes at the cost of power and a potential bias towards individuals working through reasoning items at a faster pace. With no optimal solution, we decided to report the results of all 36 items (excluding the participants who did not complete all 36 items, resulting in *n* = 164) in detail and mention any noteworthy differences from 22-item (*N* = 210), 27-item (*n* = 206), and 33-item (*n* = 185) analyses. Additionally, we included the analyses of data with the other cut-offs in the Supplementary Materials. The overall pattern of results remains the same.

**Figure 3:**
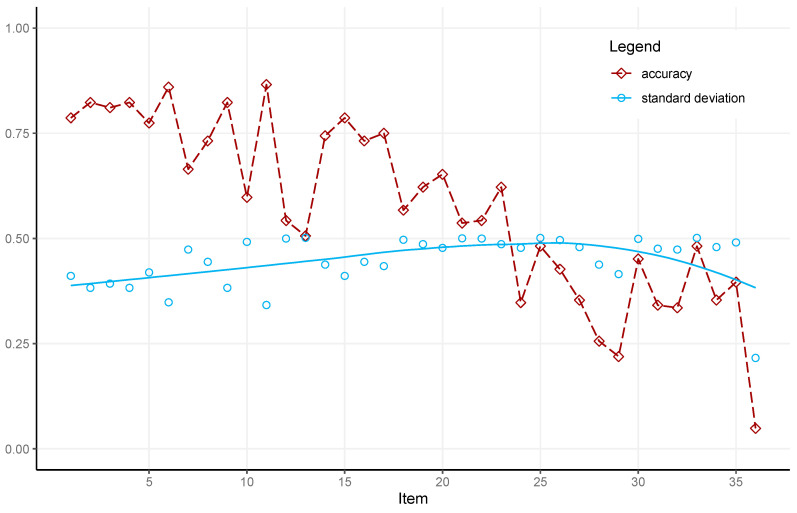
Difficulty as the mean of correct responses (P_i_) and the standard deviation for all APM items. Difficulty is depicted by the dashed line and squares. Circles and the solid polynomial regression depict standard deviation.

The means and standard deviations (*SD*) of correct responses to the APM items for the 164 participants who completed all 36 items are presented in Figure 3. Item difficulty and *SD* increased throughout the test. The mean accuracy across all 36 items of the sample was 20.65, with a standard deviation of 7.49. McDonald’s Omega for the APM responses in the analyzed sample was good (ω = 0.91). The mean test score of the current sample is comparable with the results by Hamel and Schmittmann (2006), where the participants completed all 36 items with a time limit of 20 min (mean = 20.52, *SD* = 3.87).

**Figure 4:**
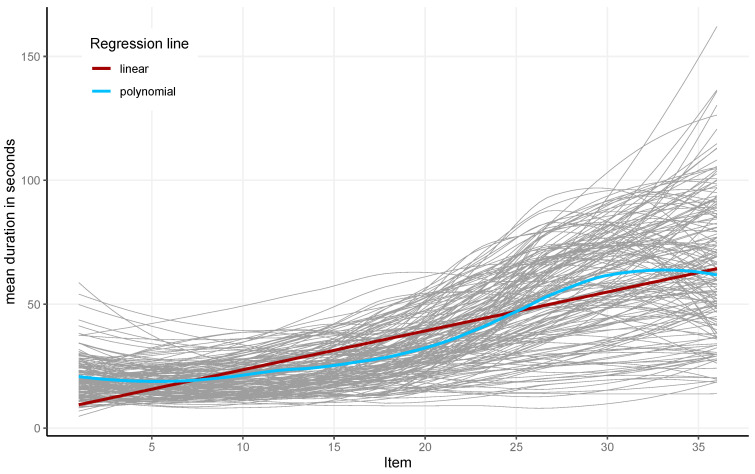
The mean item latency for all 36 items and 164 participants (gray) and the linear (red) and local polynomial (blue) regression lines for the analyzed sample.

On average, participants responded to the 36 items within 16.38 min (*SD* = 4.02 min), indicating that most participants finished the 36 items clearly below the set time limit of 30 min. Item latencies increased from the first to the twenty-seventh item (red line in Figure 4). Fitting a local polynomial regression (blue line) showed stable latencies for the first items and then an increase around approximately Item 12. This increase in item latencies somewhat flattened out around Item 30, and they even decreased for the last few items.

**Figure 5:**
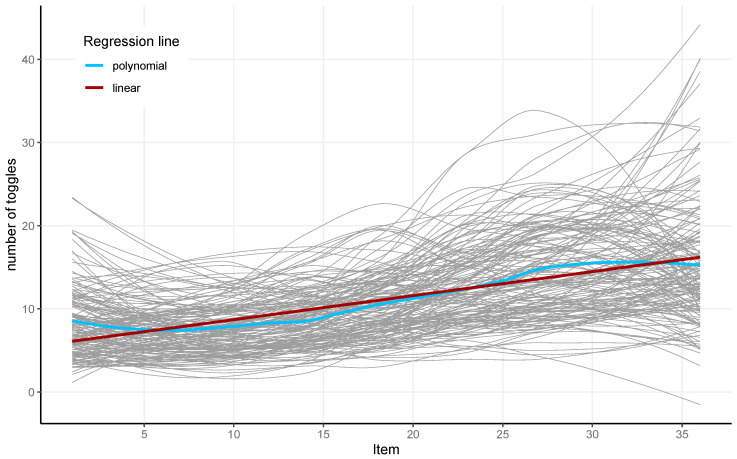
The absolute number of toggles for 36 items and 164 participants (gray) and the linear (red) and local polynomial (blue) regression lines for the analyzed sample.

The absolute number of toggles increased from item to item, as depicted by the linear regression (in red) in Figure 5. The local polynomial regression (in blue) showed a slight drop in absolute numbers of toggles for the last few items.

**Figure 6:**
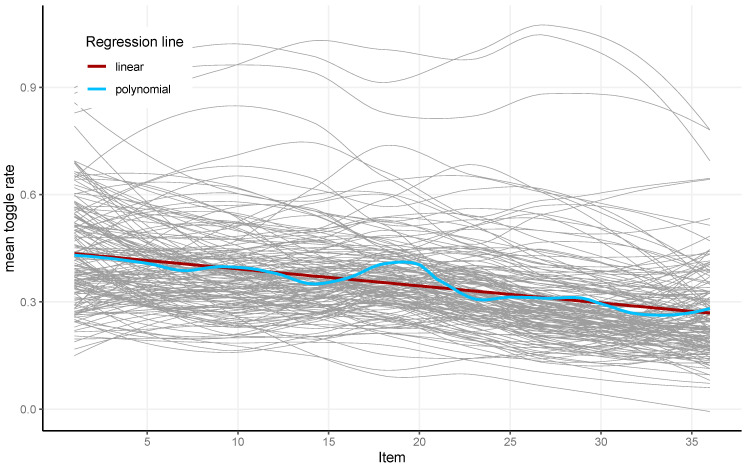
The toggle rate for each of the 36 items and 164 participants (gray) and the linear (red) and local polynomial (blue) regression lines for the analyzed sample.

The absolute number of toggles divided by the respective item latency resulted in the analyzed toggle rate. A slight decrease in the toggle rate during test completion was observed (see Figure 6). A decrease in toggle rate suggests less response elimination and more constructive matching towards the end of the test. The toggle rate had high reliability (McDonald’s Omega = 0.97).

**Figure 7:**
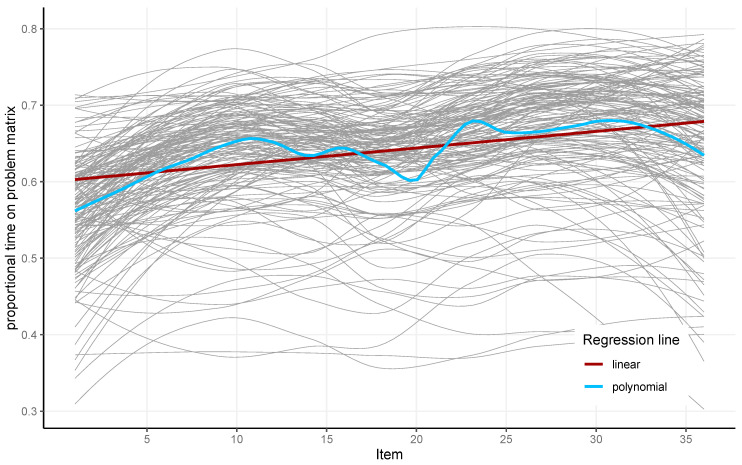
The proportional time spent on the problem matrix for each of the 36 items and 164 participants (gray) and the linear (red) and local polynomial (blue) regression lines for the analyzed sample.

The proportional time on the problem matrix was calculated by dividing the summarized duration of all fixations on the problem matrix by the total time a participant spent on the respective item. Throughout the completion of the APM, a slight increase in the proportional time on the problem matrix was observed (see Figure 7). This indicates that participants spent more time inspecting the problem matrix as the test progressed. Spending proportionally more time on the problem matrix compared to the total item latency is in line with more constructive matching. The proportional time on the problem matrix had high reliability (McDonald’s Omega = 0.97).

**Figure 8:**
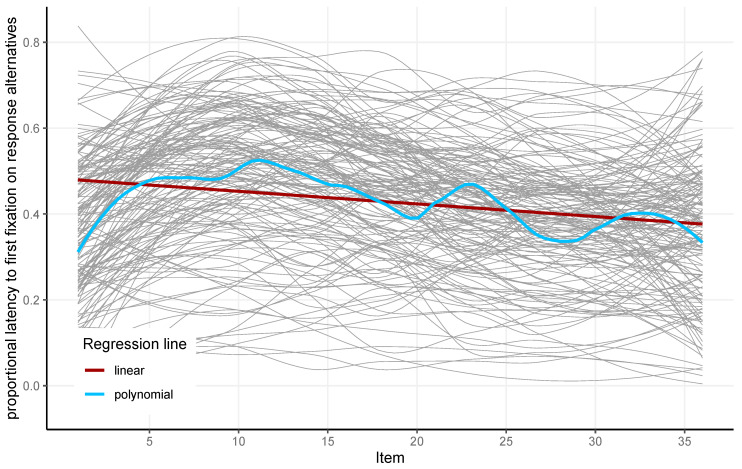
Proportional latency to the first fixation on response alternatives for each of the 36 items and 164 participants (gray) and the linear (red) and local polynomial (blue) regression lines for the analyzed sample.

The proportional time to the first fixation on response alternatives refers to the time point of the first fixation a participant made on the response alternatives while solving an item, divided by item latency. Figure 8 shows that, in relation to item latency, this point in time came earlier and earlier as the test progressed. This indicated that participants spent less time looking at the problem matrix as the test progressed before looking at the response alternatives, which is indicative of less constructive matching from item to item. The proportional time to the first fixation on response alternatives had high reliability (McDonald’s Omega = 0.94). For all the eye-tracking metrics presented and the APM scores, a correlation matrix is given in Table 1.

All of the calculated models that were used to determine a good measurement model for the toggle rate, proportional time on the problem matrix, latency to first fixation, and APM responses are summarized in Table 2. The one-factor model and a bifactor model with a linear increase for the second latent variable reflecting the item-position effect are also illustrated in Figure 2.

To investigate whether an item-position effect was present in the APM responses, three different models were fit to the data: a one-factor model (Model A); a bifactor model, where one latent variable depicted reasoning ability and the second latent variable with linearly (Model B); or quadratically (Model C) increasing factor loadings, depicting the item-position effect.

Comparing the respective models in Table 2, the bifactor model for the APM responses with a linear increase for the item-position effect (Model B) showed a better fit compared to the one-factor model (smaller AIC and larger CFI). The AIC was smaller for Model B compared to Model C. Therefore, Model B exhibited a better fit for the APM responses. Both latent variables explained a significant portion of variance (reasoning: φ = 0.315, *z* = 3.276, *p* = 0.001; item-position effect: φ = 0.397, *z* = 4.929, *p* < 0.001). The reliability was good for the reasoning (ω = 0.87) and mediocre for the item-position effect latent variable (ω = 0.65).

To investigate individual differences in the applied test-taking strategy, the same three measurement models were fit to the eye-tracking metrics. For all three eye-tracking metrics, the one-factor model led to an acceptable data description (except for the CFIs of the models for the toggle rate and the proportional time to first fixation on response alternatives), but the bifactor models described the data better, as indicated by the lower AIC and a notably higher CFI.

Among the bifactor models for the toggle rate and proportional time in the problem matrix, Model C, with the quadratically increasing factor loadings, had a lower AIC and a higher CFI than Model B, indicating that Model C described the data better. Both latent variables in Model C explained a significant portion of variance (basic toggle rate: φ = 0.011, *z* = 2.449, *p* = 0.014; change in toggle rate: φ = 0.003, *z* = 3.656, *p* < 0.001; basic proportional time on matrix: φ = 0.011, *z* = 2.449, *p* = 0.014; change in proportional time on matrix: φ = 0.003, *z* = 3.656, *p* < 0.001). Reliability was good for the basic toggle rate (ω = 0.96) and low for the change in toggle rate (ω = 0.40). Reliability for the latent variable depicting the basic proportional time on the problem matrix was good (ω = 0.96) and mediocre for the change in proportional time on the problem matrix (ω = 0.69).

For the proportional time to the first fixation on response alternatives, Model B, with linearly increasing factor loadings, described the data better, as indicated by a lower AIC and higher CFI. Both latent variables in Model B explained a significant portion of variance (basic proportional time to first fixation on response alternatives: φ = 0.011, *z* = 3.494, *p* < 0.001; change in proportional time to first fixation on response alternatives: φ = 0.045, *z* = 4.961, *p* < 0.001). Reliability for the latent variable depicting the basic proportional time to first fixation on response alternatives was good (ω = 0.91) and acceptable for changes in proportional time to first fixation on response alternatives (ω = 0.71).

To investigate the interplay of latent variables extracted from the APM responses and the latent variables extracted from the eye-tracking metrics, the better-fitting measurement models from each were combined, respectively (last three rows in Table 2). This included Model B for APM responses and proportional time to the first fixation on response alternatives and Model C for the toggle rate and the proportional time on the problem matrix.

All models showed an acceptable fit except for the CFI. According to Kenny et al. (2015), the CFI is not informative when the RMSEA of the baseline model is <0.158. This was the case for all three models (toggle rate, 0.110; proportional time on matrix, 0.111; proportional time to first fixation on response alternatives, 0.088). Hence, the CFI was not further used to determine the model’s fit.

**Figure 9:**
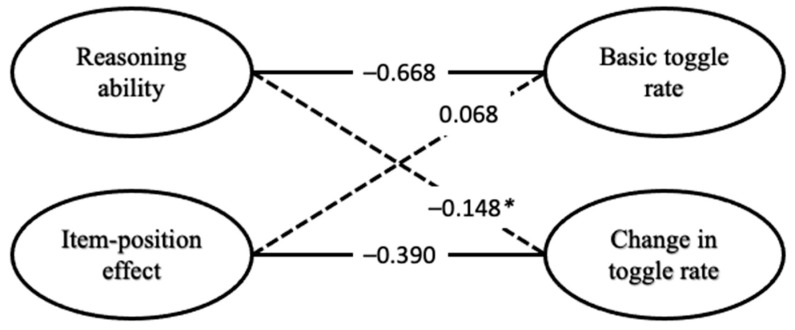
Correlations between latent variables of the full model regarding toggle rate. Solid lines indicate significant correlations, and dashed lines are non-significant ones. * For the 33-item cut-off, where only 33 items and the participants who completed all 33 items were used to calculate the full model, this correlation was significant *r* = −0.256 (*p* = 0.018). For all other cut-offs (see Supplementary Materials), the pattern of results was as depicted in this figure.

Correlations of the latent variables of the full model containing APM responses and toggle rate are depicted in Figure 9. The latent variable for reasoning ability was correlated with the latent variable representing the basic toggle rate (*r* = −0.668, *p* = 0.003) but not with the latent variable representing the change in toggle rate (*r* = −0.148, *p* = 0.193). The item-position effect in APM responses was significantly correlated with the latent variable representing the change in toggle rate (*r* = −0.390, *p* = 0.012) but not with the latent variable representing the basic toggle rate (*r* = −0.068, *p* = 0.414).

**Figure 10:**
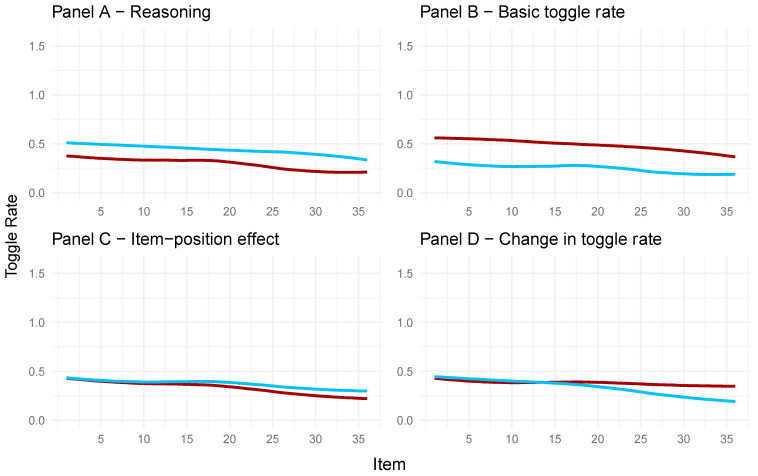
Toggle rate for participants with high or low values on the latent variables representing reasoning (**Panel A**), the basic toggle rate (**Panel B**), the item-position effect (**Panel C**), and the change in toggle rate (**Panel D**). Blue lines represent participants with low factor scores on the latent variable. Red lines represent participants with high factor scores on the depicted latent variable.

To illustrate and better understand these relations, descriptive graphics were created (see Figure 10). For the four panels in Figure 10, the factor scores of the full model regarding the toggle rate and APM responses were extracted. For each panel and the respective latent variable (Panels A to D), the 50 highest and 50 lowest factor scores were taken to create the distinction between participants with high and low manifestations on the latent variable. Therefore, the toggle rate throughout the test is illustrated in the graphs, with separate polynomial regression lines used for low and high manifestations on the given latent variable.

In Panel A of Figure 10, the observed toggle rates for participants with high factor scores on the latent variable of reasoning ability (red line) and participants with low factor scores on reasoning ability (blue line) are depicted. Participants with higher factor scores on the reasoning ability variable showed a lower toggle rate for each item compared to participants with low reasoning ability factor scores. This means participants rather engaged in response elimination when their reasoning ability was low. This is in line with Vigneau et al.’s (2006) conclusion.

Panel B in Figure 10 shows that the observed toggle rate in the APM items was higher for participants with high factor scores on the latent variable representing the basic toggle rate (red line) compared to participants with low factor scores (blue line). This underlines that the latent variable for the basic toggle rate indeed reflects a general toggle rate for each participant throughout the test.

As can be taken from Panel C in Figure 10, participants with low (blue line) and high (red line) factor scores on the item-position effect in the APM responses reduced their toggle rate throughout the APM, reflecting a change in strategy. This change seemed larger for participants with high factor scores on the item-position effect^1^, indicating that participants with a more pronounced item-position effect adapt their strategy more during test taking and shift to making fewer toggles; that is, less response elimination and more constructive matching compared to participants with a less pronounced item-position effect.

Participants with high factor scores (red line) on the change in toggle rate (Panel D, Figure 10) showed no explicit change in their overall toggle rate. Participants with low values (blue line), on the other hand, showed a decrease in their overall toggle rate throughout the test. This means that a low manifestation on the latent variable depicting the change in toggle rate is indicative of a decrease in toggle rate, i.e., more constructive matching as the test progresses.

These results are also reflected in the correlations of the full model, albeit less descriptive (Figure 9). Low values in the change in toggle rate were related to high values on the item-position effect in the APM responses. Low values in reasoning ability were related to high values in the basic toggle rate.

**Figure 11:**
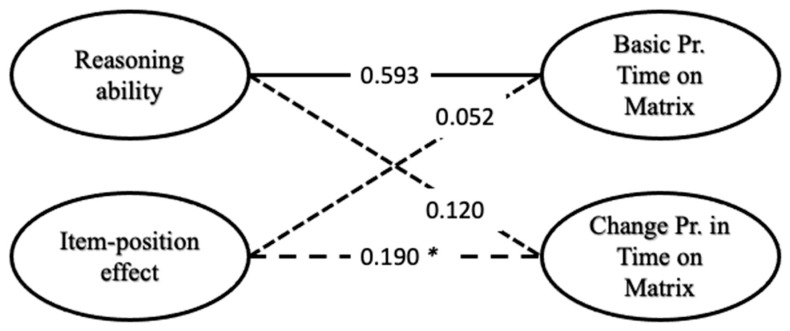
Correlations between latent variables of the full model regarding the proportional time on the problem matrix. Solid lines indicate significant correlations, and dashed lines are non-significant ones. * For the 27-item cut-off, where only 27 items and the participants who completed all 27 items were used to calculate the full model, this correlation was significant *r* = 0.529 (*p* = 0.004).

In the model combining the models regarding proportional time on the problem matrix and the APM responses (Figure 11), a higher reasoning ability was significantly correlated with the latent variable representing the basic time spent on the problem matrix (*r* = 0.593, *p* = 0.001). This is in line with the previous findings of higher reasoning ability being related to constructive matching (e.g., Jastrzębski et al. 2018), as proportionally, more time spent on the problem matrix is the basis for more constructive matching. The correlation between the latent variable depicting the item-position effect and the change in proportional time on the problem matrix was not significant (*r* = 0.190, *p* = 0.133).

**Figure 12:**
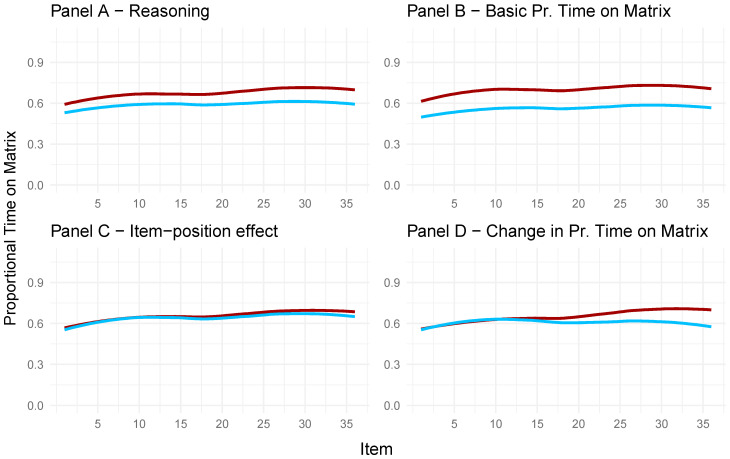
Proportional time on the problem matrix for participants with high or low values on the latent variables representing reasoning (**Panel A**), the basic time on the problem matrix (**Panel B**), the item-position effect (**Panel C**), and the change in time on the problem matrix (**Panel D**). Blue lines represent participants with low factor scores on the latent variable. Red lines represent participants with high factor scores on the depicted latent variable.

The panels in Figure 12 were created to display the proportional time spent on the problem matrix for participants with high (red lines) and low (blue lines) factor scores on the respective latent variables depicted separately in each panel. Panel A in Figure 12 depicts the proportional time spent on the problem matrix for individuals with high (red) and low (blue) factor scores on the latent reasoning variable. In line with previous research, more time spent on the problem matrix (i.e., constructive matching) coincided with a higher reasoning ability.

Panel B shows that participants with high factor scores on the latent variable depicting the basic proportional time on the matrix spent proportionally more time on the problem matrix compared to the participants with low factor scores on the latent variable. Hence, proportionally, more time spent on the problem matrix coincides with high factor scores on the latent variable, depicting basic proportional time on the matrix (Panel B) and high factor scores on the latent reasoning variable (Panel A). This is reflected in the strong positive correlation between the two latent variables, depicting reasoning ability and basic time on the problem matrix (Figure 11).

In Panel C, no differences between individuals with high and low factor scores on the latent variable reflecting the item-position effect can be seen, as participants with high (red) and low (blue) factor scores on the item-position effect of the APM responses did not differ regarding the proportional time spent on the problem matrix.

In Panel D of Figure 12, the difference between participants with high (red) and low (blue) factor scores on the latent variable for the change in proportional time spent on the problem matrix is visible for the last 10 items. This indicated that participants with high factor scores on the latent variable depicting the change in proportional time spent on the problem matrix spent more time on the problem matrix for the last 15 items compared to the participants with low factor scores on the said latent variable.

**Figure 13:**
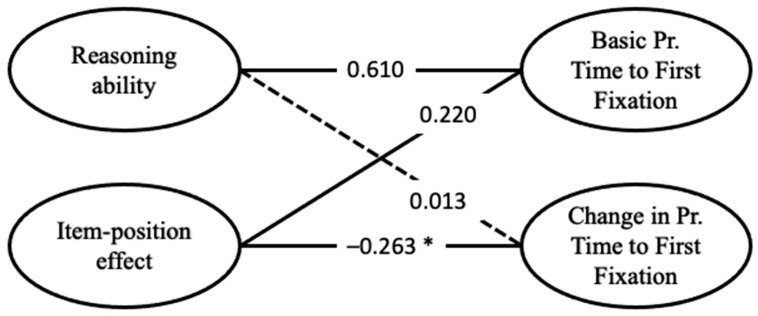
Correlations between latent variables of the full model regarding the proportional time to first fixation on response alternatives. Solid lines indicate significant correlations, and dashed lines are non-significant ones. * For the 27-item and 22-item cut-offs, this correlation was not significant.

Regarding the full model, including the APM responses and the proportional time to the first fixation on response alternatives, correlations are given in Figure 13. As with the other two eye-tracking metrics, the latent reasoning variable exhibited a noteworthy positive correlation, with the latent variable capturing the basic proportional time to first fixation on the response alternatives (*r* = 0.610, *p* < 0.001). Further, the item-position effect was negatively associated with the change in this eye-tracking metric during test-taking (*r* = −0.263, *p* = 0.019). The negative correlation indicated that a pronounced item-position effect coincided with low factor scores on the latent variable, depicting the change in proportional time to the first fixation on response alternatives. Additionally, the latent variable depicting the basic proportional time to first fixation on response alternatives showed a small correlation with the latent variable of the item-position effect (*r* = 0.220, *p* = 0.029). This indicates that participants with a pronounced item-position effect also show high values in basic proportional time to the first fixation on response alternatives. Hence, these individuals spent more time investigating the problem matrix before turning to the response alternatives, indicative of constructive matching.

**Figure 14:**
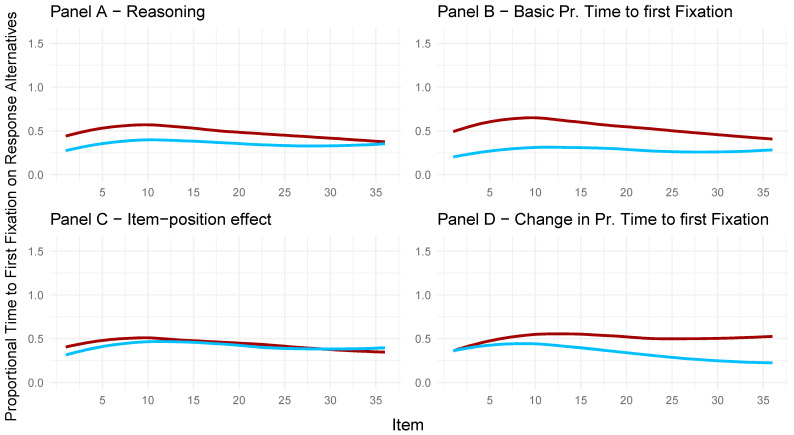
Proportional time to first fixation on response alternatives for participants with high or low values on the latent variables representing reasoning (**Panel A**), the basic proportional time to first fixation on response alternatives (**Panel B**), the item-position effect (**Panel C**), and the change in proportional time to first fixation on response alternatives (**Panel D**). Blue lines represent participants with low factor scores on the latent variable. Red lines represent participants with high factor scores on the depicted latent variable.

The proportional time to the first fixation on response alternatives is illustrated in more detail regarding the different latent variables in the full model in Figure 14. The proportional time to first fixation on response alternatives is displayed for participants with the 50 highest (red line) and 50 lowest (blue line) factor scores on the respective latent variable. Panel A shows that participants with the highest factor scores on the latent reasoning variable had a longer proportional time to their first fixation on response alternatives than the participants with the lowest factor scores on the latent reasoning variable. Hence, participants with a higher reasoning ability spent proportionally more time investigating the problem matrix before turning to the response alternatives compared to the participants with a lower reasoning ability. However, this difference became smaller from item to item and disappeared for the last few items.

Panel B shows a similar picture. The difference in proportional time to first fixation on response alternatives between participants with the 50 highest and 50 lowest factor scores on the latent variable depicting the basic proportional time to first fixation on response alternatives became smaller as the test progressed.

Regarding the latent variable depicting the item-position effect, Panel C in Figure 14 shows that the 50 participants with particularly high factor scores cannot be distinguished from the 50 participants with low factor scores on the latent item-position effect variable regarding their proportional time to first fixation on response alternatives.

Panel D highlights that participants with high factor scores in the latent variable depicting a change in proportional time to the first fixation on response alternatives did not change their proportional time to their first fixation on the response alternatives as the test progressed. Participants with low factor scores, on the contrary, exhibited decreasing values, hence progressively engaged in less constructive matching as the test progressed, since they spent proportionally less time investigating the problem matrix before inspecting the response alternatives.

## 4. Discussion

The present study had three primary objectives. The first was to investigate the presence of an item-position effect in APM responses. Consistent with prior research (e.g., Ren et al. 2014; Schweizer et al. 2012), a bifactor model—accounting for both reasoning ability and an item-position effect—provided a superior fit to the present data compared to a one-factor model. This confirms the notion that as individuals progress through the APM, additional variance emerges that cannot be attributed solely to reasoning ability.

The second objective was to explore whether individual differences in strategy use and any shifts therein could be captured at a latent level using eye-tracking metrics within a bifactor modeling framework. All three eye-tracking indicators—toggle rate, proportional time on the problem matrix, and proportional time to first fixation on response alternatives—showed a substantial change from the first to the last item, as illustrated in Figure 6, Figure 7 and Figure 8. However, the toggle rate decreased, and the proportional time on the problem matrix increased during the test, indicating that participants adapted their strategy use to more constructive matching. The decreasing proportional time to first fixation on response alternatives, however, suggested that participants used more response elimination as the test progressed.

Given the change in all three eye-tracking metrics, it was not surprising that individual differences could be better explained by bifactor models rather than one-factor models. Such bifactor models account for rather static individual differences across the test (e.g., a basic toggle rate) and a statistically separable increase in differences between individuals as the test progresses (e.g., a change in the toggle rate). More specifically, the latent variables with increasing factor loadings depict increasing individual differences from the first to the last item, indicating that differences between individuals grow from item to item regarding their strategy use. The individual differences in the changes observed in Figure 6, Figure 7 and Figure 8 are represented in these latent variables.

The third objective investigated whether reasoning ability and the item-position effect were related to strategy use and changes therein. Across all eye-tracking metrics, a higher reasoning ability was associated with more constructive matching. This aligns with prior findings that link a higher cognitive ability with (more) constructive matching (Gonthier and Roulin 2020; Jastrzębski et al. 2018; Vigneau et al. 2006). However, reasoning ability did not correlate with the latent variables representing a change in the eye-tracking metrics during test taking^2^. This result suggests that the tendency to adopt the strategy used during test taking is independent of reasoning ability.

The results regarding the relationship between the item-position effect and changes in strategy use were somewhat heterogeneous and depended on the operationalization of strategy use. The item-position effect correlated positively with the change in toggle rate but negatively with the change in the proportional time to the first fixation on response alternatives. This indicates that the item-position effect came along with an increase in constructive matching when operationalized by the toggle rate, but with a decrease in constructive matching when operationalized by the proportional time to first fixation on response alternatives. For the proportional time on the problem matrix, no systematic relationship with the item-position effect could be observed. (It should be noted, however, that a positive correlation between the item-position effect and the change in the proportional time spent on the problem matrix emerged for the cut-off at item 27; see Supplementary Materials).

These results seem to contradict but foremost highlight the possibility that there could be different stages in problem-solving behavior. The contemporary interpretation of a continuous spectrum of strategy use ranging from response elimination towards constructive matching is likely ill-equipped to account for such nuanced problem-solving behavior. This is emphasized by the finding that the toggle rate in the current data indicated an increase, while proportional time to first fixation on response alternatives indicated a decrease in constructive matching as the test progressed. Early fixation on response alternatives focuses on the early stages of problem-solving behavior and possibly captures the individual trying to obtain an overview of the broader problem before investigating the problem matrix in more detail. Therefore, it is possible that, while the proportional time to the first fixation on response alternatives especially accounts for the early processing stage, the toggle rate covers the whole problem-solving behavior, including the first to the last fixation on response alternatives. Hence, the toggle rate includes behavior possibly specific to early stages of problem-solving behavior (e.g., trying to obtain an overview of the problem), actual problem-solving behavior, and possibly an additional final stage of problem-solving behavior (e.g., double-checking the answer before submitting a response). Hence, the discovered discrepancies between the results of the current study highlight the possibility of different stages within problem-solving behavior. Future studies could attempt to account for the specific sequence of eye movement, as in, for example, Hayes et al. (2015), and then investigate whether a specific pattern is more likely to lead to an adaption in solving behavior on difficult items.

Another unexpected finding was a significant correlation between the item-position effect and the basic level of proportional time to first fixation on response alternatives—a pattern not mirrored in the other eye-tracking metrics. This metric also showed no correlation with item latency despite all three metrics being adjusted for latency. Prior to adjustment, the three metrics shared different degrees of variance with item latency (Table 1: the toggle rate showed the weakest relationship (*r* = 0.415), time on the problem matrix was the strongest (*r* = 0.951), and the time to first fixation on response alternatives fell in between (*r* = 0.567)). These differences may also point to different stages in problem-solving behavior and could explain the inconsistent results across metrics and warrant further examination of item latency effects.

**Figure 15:**
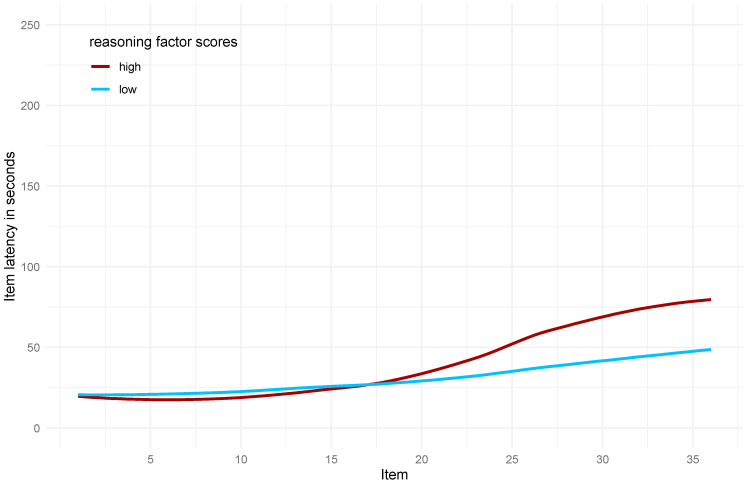
Item latencies for participants with high or low reasoning ability factor scores. Note. Given are the item latencies in seconds and a polynomial trend line for the participants, with the 50 highest (in red) and 50 lowest (in blue) reasoning ability factor scores of the full model.

Visualization of item latency patterns revealed that participants with higher reasoning ability showed a steeper increase in response time for the more difficult items (from item 17 onwards) compared to those with lower ability (Figure 15). This divergence persisted until the final items. These findings replicate the results of Cheyette and Piantadosi (2024), who reported that, on difficult APM items, the best-performing participants spent about three times as long as the lowest-performing participants. Their results indicate that with increasing reasoning ability, participants modulated their item latencies to a larger degree for difficult items. According to Perret and Dauvier (2018), this is also the case in children. In Cheyette and Piantadosi (2024), this modulation was gradually smaller in individuals with a lower reasoning ability. Participants with the lowest reasoning scores even seemed to spend less time on difficult items compared to easy items.

Time pressure may play a critical role in shaping such response patterns. Gonthier (2023) showed that high-ability individuals modulate item latency less under high time pressure, whereas low or no pressure permits more adaptive timing. In the current study, the three unadjusted eye-tracking metrics exhibited different correlations with the APM test score (number of toggles, *r* = −0.159; time on matrix, *r* = 0.634; latency to first fixation on response alternatives, *r* = 0.440). This highlights that while the three metrics can be used as indicators of strategy use, they seem to be connected differently to reasoning ability, supporting the interpretation that strategy use, item latency, and reasoning ability interact in complex ways. Additionally, the results of Gonthier (2023) highlight how time limits differentially impact participants depending on the item’s difficulty and individual reasoning capacity. Hence, it is unclear to what extent the implemented time limit might have influenced item latencies and strategy use in the current study. This limits the generalizability of the present results to reasoning tests administered without a time limit.

Nevertheless, it is interesting that around item 17—the same point at which item latencies began to diverge between ability groups—differences in strategy use also emerged. Participants with strong item-position effects reduced the toggling rate but also showed a proportionally earlier fixation on response alternatives, suggesting a shift in strategy. In contrast, those with weaker item-position effects showed no adaptation.

Liu et al. (2023) found that high-ability participants increased their use of constructive matching on difficult items, while medium-ability participants maintained a stable level, and low-ability participants decreased their use. They also reported that item difficulty alone did not predict constructive matching, but its interaction with reasoning ability did. By contrast, Jarosz et al. (2019), using think-aloud protocols, observed a general decline in constructive matching as item difficulty increased. However, the interaction effect of working memory capacity and item difficulty was not significant in their study. Nevertheless, they found that a higher working memory capacity predicted a higher likelihood of employing constructive matching at the beginning of the test, possibly explaining the mediating role of strategy use regarding working memory capacity and APM performance.

Gonthier and Roulin (2020), using questionnaire data, similarly reported a decrease in constructive matching as the test progressed, modulated by working memory capacity and motivational factors such as the need for cognition. Their findings imply that individuals with higher cognitive and motivational resources were more likely to maintain constructive matching on difficult items, while others shifted toward less demanding strategies.

These varying results illustrate the difficulty in theorizing about strategy shifts. Differences in methodology (e.g., eye-tracking, self-reports, verbal protocols), APM implementations (e.g., full vs. short versions), and testing conditions (e.g., time limits) may all contribute. Notably, Liu et al. (2023) reported an interaction between ability and item difficulty, while Jarosz et al. (2019) found no such interaction. The current study also suggests that strategy adaptation may be more strongly tied to the item-position effect than to reasoning ability per se, as no significant correlations were found between reasoning ability and changes in the eye-tracking metrics.

This interpretation aligns with findings that constructive matching can be enhanced through intervention (Gonthier and Thomassin 2015) and is associated with rule knowledge (Loesche et al. 2015). Studies show that participants apply constructive matching more often when they are familiar with item rules, suggesting that rule learning facilitates this strategy. As most reasoning tests rely on a limited number of rule types (Carpenter et al. 1990), mastery of these rules would enable more efficient problem-solving.

Supporting this, studies have linked the item-position effect in reasoning ability measures with rule learning (Ren et al. 2014; Schweizer et al. 2021). Experimental work by von Gugelberg et al. (2025) showed that introducing new rules after repeated exposure disrupted the item-position effect, further substantiating the rule-learning hypothesis regarding the item-position effect.

Constructive matching requires deeper engagement with the problem matrix, involving systematic rule analysis (e.g., Snow 1978). This process likely promotes rule acquisition and supports the continued use of constructive matching. Thus, the observed negative correlation between the item-position effect and change in toggle rate may reflect this interplay between rule learning and strategy adaptation.

However, when analyzing the proportional time to the first fixation on response alternatives, the fixation on response alternatives happened earlier and earlier as the test progressed for participants with a strong manifestation of the item-position effect. This translates to participants spending less time investigating the problem matrix before consulting the response alternatives, as would be typical for response elimination. Hence, in the full model regarding the toggle rate, the item-position effect is associated with behavior assumed to depict more constructive matching, while in the full model regarding proportional time to first fixation on response alternatives with solving behavior is presumably related to less constructive matching. Therefore, it seems likely that the different eye-tracking metrics capture different aspects of the solving process and possibly tap into different abilities and other factors impacting solving behavior to a different degree as item difficulty increases throughout the test.

Unfortunately, the current study design does not allow for a clear distinction between item-position effect and item difficulty, as the APM items are arranged in ascending order of difficulty. While prior studies have shown these effects to be distinct (e.g., Schweizer and Troche 2018; Zeller et al. 2017), their overlap in the current data makes disentanglement difficult. Future research should consider presenting APM items in a pseudo-random order to isolate these factors.

Such designs could also help clarify inconsistent findings regarding constructive matching. While in Liu et al. (2023), an increase was observed, other studies reported a decrease as the test progressed (Jarosz et al. 2019; Gonthier and Roulin 2020), and the current study found both. It remains unclear whether motivation, disengagement (e.g., Nagy et al. 2023), or cognitive resources drive these divergent patterns. These variables may interact with both item difficulty and the item-position effect.

Another issue involves the shape of change captured by the second latent variable in the bifactor models. There is no clear consensus on whether a linear or quadratic function better describes these changes in reasoning ability measures (Schweizer et al. 2009). In this study, a linear increase provided a better fit for the APM responses across all cut-offs. For eye-tracking metrics, the trajectory varied: the proportional time to first fixation on response alternatives was better modeled linearly, while the toggle rate and time on the matrix followed a quadratic pattern. This suggests the former captures smaller individual differences in later test items, possibly due to its reliance on a single time-based value per item and constraints imposed by the time limit. Future research should explore different functional forms in bifactor models and seek to experimentally elicit specific patterns.

Finally, this study cannot fully untangle the complex relationships between reasoning ability, strategy use, and item characteristics. Previous studies reported different directions regarding changes in strategy use in the APM, with reasoning ability being a valid predictor of constructive matching (Laurence et al. 2018). Further personality factors, such as the need for cognition (Gonthier and Roulin 2020) or narcissism (Birney et al. 2017), could be mediating factors regarding a possible interaction of ability and item difficulty. With the rule-based nature of APM items, it also seems plausible that the ability to learn rules impacts strategy use, yet an experimental design to directly test the assumption will be needed to draw more solid conclusions.

### Limitations and Outlook

Although time limits are generally deemed acceptable for maintaining test validity (Hamel and Schmittmann 2006), evidence from Gonthier (2023) suggests that such constraints can bias both item latency and strategy use. In his study, the 10-min time limit reduced constructive matching across all items but not for the 20-min time limit version compared to the untimed version. Further, all versions showed a rather similar decline in constructive matching as the test progressed (the decline for the 20-min version was slightly smaller compared to the other versions). In contrast, the current study observed increasing constructive matching as the test progressed, but additionally, a decrease in proportional time to first fixation on response alternatives. Whether this pattern would persist under stricter time constraints or without a time limit remains unknown. This further highlights the need for a replication of results in an untimed test setting.

It is also important to mention that although constructive matching and response elimination are the most distinct strategies, some studies have identified a third strategy in questionnaire data (Li et al. 2022) or think-aloud protocols (Jarosz et al. 2019). It is likely that this third strategy is somewhat of a mix of the two. Nevertheless, it highlights that the strategies assessed here are better understood as continuous dimensions describing the extent or degree of a participant engaging in a strategy rather than binary categories.

To better understand strategy use, future research could integrate cognitive measures such as planning ability (Laurence and Macedo 2023) or cognitive control (Braver 2012; von Gugelberg et al. 2021). Motivational factors should also be considered, as they are known to affect performance, even in low-stakes testing contexts. For example, a detailed investigation of disengagement and rapid guessing (e.g., Nagy et al. 2023) could be an interesting avenue to explore. Moreover, the need for cognition could also moderate strategy use and should be assessed alongside other individual differences (Gonthier and Roulin 2020).

Finally, the meta-reasoning framework (Ackerman and Thompson 2017) may offer an additional and valuable perspective. It posits that individuals may strategically opt out of answering when problem-solving seems unfeasible. Law et al. (2022) found factorial stability for such giving-up or opting-out tendencies. While the tendency to give up showed a moderate correlation with reasoning ability in Law et al. (2022), it would be interesting to investigate the effects of such tendencies on item latencies, strategy use, and whether this adaptive behavior is related to the item-position effect.

In summary, this study reinforces the view that performance in reasoning tests not only reflects reasoning ability but also dynamic processes, including individual differences in ad-hoc learning, strategy adaptation, or even different stages within problem-solving behavior. The item-position effect may serve as a valuable marker of such processes and deserves closer theoretical and empirical attention, as in the current study, changes in strategy use during test taking were more consistently related to the item-position effect than reasoning ability itself. Integrating psychometric modeling with methods like eye tracking, augmented by, for example, think-aloud protocols, offers a promising avenue for uncovering the cognitive mechanisms underlying problem-solving behavior and reasoning test performance.

## Figures and Tables

**Table 1:** The correlation matrix of the APM test scores and eye-tracking metrics for the 164 participants completing all 36 items on the APM.

	APM	Tog Rate	N of Tog	I-Latency	T on M	T on RA	FF on RA	Pr. T on M	Pr. T on RA	Pr. T to FF
APM—Score	APM	0.000	0.042	0.000	0.000	0.250	0.000	0.000	0.000	0.000
Toggle Rate	−0.613	Tog Rate	0.000	0.000	0.000	0.813	0.000	0.000	0.000	0.000
Number of Toggles	−0.159	0.471	N of Tog	0.000	0.001	0.000	0.011	0.001	0.000	0.000
Item Latency	0.546	−0.502	0.415	I-Latency	0.000	0.000	0.000	0.000	0.000	0.979
Time on Matrix	0.634	−0.583	0.266	0.951	T on M	0.000	0.000	0.000	0.000	0.057
Time on Response Alternatives	*0.090*	*0.019*	0.716	0.652	0.557	T on RA	0.048	0.686	0.000	0.000
Latency to First Fixation on RA	0.440	−0.709	−0.199	0.567	0.633	0.154	FF on RA	0.000	0.000	0.000
Proportional Time on Matrix	0.546	−0.650	−0.246	0.361	0.601	*0.032*	0.536	Pr. T on M	0.000	0.000
Proportional Time on Response Alternatives	−0.455	0.772	0.317	−0.423	−0.485	0.276	−0.624	−0.561	Pr. T on RA	0.000
Proportional Time to First Fixation on RA	0.482	−0.691	−0.704	*0.002*	*0.149*	−0.408	0.650	0.522	−0.565	Pr. T to FF

Note. Given correlations (non-significant values are *italic*). Abbreviations in the first row and diagonally across the matrix correspond to information given in the first column. The APM test score was calculated as the number of correct answers to the 36 items.

**Table 2:** Goodness-of-fit indices and criteria for each of the calculated measurement models.

	χ^2^ (*df*)	*p*	CFI	RMSEA	SRMR	AIC
APM responses						
one-factor model/Model A	745.30 (594)	<0.001	0.871	0.040	0.065	6192
**bifactor model/Model B**	**686.42 (593)**	**0.005**	**0.920**	**0.031**	**0.064**	**6137**
bifactor model/Model C	694.95 (593)	0.002	0.913	0.033	0.063	6146
Toggle rate						
one-factor model/Model A	796.19 (594)	<0.001	0.894	0.048	0.048	−6235
bifactor model/Model B *	769.63 (593)	<0.001	0.907	0.045	0.048	−6257
**bifactor model/Model C**	**770.69 (593)**	**<0.001**	**0.907**	**0.045**	**0.047**	**−6258**
Proportional Time on Matrix						
one-factor model/Model A	838.89 (594)	<0.001	0.906	0.053	0.051	−13,214
bifactor model/Model B	776.03 (593)	<0.001	0.931	0.045	0.053	−13,290
**bifactor model/Model C**	**770.35 (593)**	**<0.001**	**0.933**	**0.045**	**0.049**	**−13,299**
Proportional time to the first fixation on response alternatives						
one-factor model/Model A	738.41 (594)	<0.001	0.895	0.040	0.065	−827
**bifactor model/Model B**	**671.51 (593)**	**0.014**	**0.943**	**0.030**	**0.064**	**−894**
bifactor model/Model C ^+^	683.47 (593)	<0.001	0.934	0.032	0.063	−882
Full Models with Model B for the APM responses and the better-fitting model for the respective eye-tracking metric						
Toggle rate/ Model C	3171.08 (2478)	<0.001	0.799	0.042	0.064	−210
Proportional time on Matrix/ Model C	3343.64 (2478)	<0.001	0.807	0.047	0.068	−7219
Proportional time to the first fixation on response alternatives/Model B	3189.15 (2478)	<0.001	0.777	0.042	0.071	5107

Model A is the one-factor model. Model B includes a second latent variable with linearly increasing factor loadings, and Model C with quadratically increasing factor loadings from the first to the last item. The models in **bold** indicate the better-fitting model. * The 22-item and 27-item cut-offs favor Model B, concluding that the pattern of results in the full model does not change. ^+^ Only for the 22-item cut-off does this model exhibit an acceptable fit.

## Data Availability

The original contributions presented in this study are included in the supplementary material. Further inquiries can be directed to the corresponding author.

## Notes

1 The difference in changes in the toggle rate between participants with high and low manifestations on the latent variable of the item-position effect in APM responses is more evident in the analysis with the 22-item cut-off (Supplementary Materials—Figure S10_22, Panel C).

2 Except for the one instance in the full model regarding the toggle rate and the 33-item cut-off.

## Supplementary Materials

All Figures (S3–S15) and Tables (S1, S2) for the different cut-offs can be downloaded at: https://www.mdpi.com/article/10.3390/jintelligence13070077/s1.

## References

[B1-jintelligence-13-00077] Ackerman Rakefet, Thompson Valerie A. (2017). Meta-reasoning: Monitoring and control of thinking and reasoning. Trends in Cognitive Sciences.

[B2-jintelligence-13-00077] Bethell-Fox Charles E., Lohman David F., Snow Richard E. (1984). Adaptive reasoning: Componential and eye movement analysis of geometric analogy performance. Intelligence.

[B3-jintelligence-13-00077] Birney Damian P., Beckmann Jens F., Beckmann Nadin, Double Kit S. (2017). Beyond the intellect: Complexity and learning trajectories in Raven’s Progressive Matrices depend on self-regulatory processes and conative dispositions. Intelligence.

[B4-jintelligence-13-00077] Bojko Aga (2013). Eye Tracking, the User Experience: A Practical Guide to Research.

[B5-jintelligence-13-00077] Braver Todd. S. (2012). The variable nature of cognitive control: A dual mechanisms framework. Trends in Cognitive Sciences.

[B6-jintelligence-13-00077] Carpenter Patricia A., Just Marcel A., Shell Peter (1990). What one intelligence test measures: A theoretical account of the processing in the Raven Progressive Matrices Test. Psychological Review.

[B7-jintelligence-13-00077] Chen Fang Fang (2007). Sensitivity of goodness of fit indexes to lack of measurement invariance. Structural Equation Modeling: A Multidisciplinary Journal.

[B8-jintelligence-13-00077] Cheyette Samuel J., Piantadosi Steven T. (2024). Response to Difficulty Drives Variation in IQ Test Performance. Open Mind.

[B9-jintelligence-13-00077] DiStefano Christine, Schweizer Karl, DiStefano Christine (2016). Examining fit with structural equation models. Principles and Methods of Test Construction.

[B10-jintelligence-13-00077] Gignac Gilles E. (2015). Raven’s is not a pure measure of general intelligence: Implications for g factor theory and the brief measurement of g. Intelligence.

[B11-jintelligence-13-00077] Gonthier Corentin (2023). Should intelligence tests be speeded or unspeeded? A brief review of the effects of time pressure on response processes and an experimental study with Raven’s Matrices. Journal of Intelligence.

[B12-jintelligence-13-00077] Gonthier Corentin, Roulin Jean-Luc (2020). Intraindividual strategy shifts in Raven’s matrices, and their dependence on working memory capacity and need for cognition. Journal of Experimental Psychology: General.

[B13-jintelligence-13-00077] Gonthier Corentin, Thomassin Noémylle (2015). Strategy use fully mediates the relationship between working memory capacity and performance on Raven’s matrices. Journal of Experimental Psychology: General.

[B14-jintelligence-13-00077] Gonthier Corentin, Harma Kahina, Gavornikova-Baligand Zdenka (2024). Development of reasoning performance in Raven’s matrices is grounded in the development of effective strategy use. Journal of Experimental Psychology: General.

[B15-jintelligence-13-00077] Hamel Ronald, Schmittmann Verena D. (2006). The 20-minute version as a predictor of the Raven Advanced Progressive Matrices Test. Educational and Psychological Measurement.

[B16-jintelligence-13-00077] Hayes TaylorR., Petrov Alexander A., Sederberg Per B. (2015). Do we really become smarter when our fluid-intelligence test scores improve?. Intelligence.

[B17-jintelligence-13-00077] Jarosz Andrew F., Raden Megan J., Wiley Jennifer (2019). Working memory capacity and strategy use on the RAPM. Intelligence.

[B18-jintelligence-13-00077] Jastrzębski Jan, Ciechanowska Iwona, Chuderski Adam (2018). The strong link between fluid intelligence and working memory cannot be explained away by strategy use. Intelligence.

[B19-jintelligence-13-00077] Kenny David A. (2015). Measuring Model Fit. https://davidakenny.net/cm/fit.htm.

[B20-jintelligence-13-00077] Laurence Paulo G., Macedo Elizeu C. (2023). Cognitive strategies in matrix-reasoning tasks: State of the art. Psychonomic Bulletin & Review.

[B21-jintelligence-13-00077] Laurence Paulo G., Mecca Tatiana P., Serpa Alexandre, Martin Romain, Macedo Elizeu C. (2018). Eye Movements and Cognitive Strategy in a Fluid Intelligence Test: Item Type Analysis. Frontiers in Psychology.

[B22-jintelligence-13-00077] Law Marvin K., Stankov Lazar, Kleitman Sabina (2022). I choose to opt-out of answering: Individual differences in giving up behaviour on cognitive tests. Journal of Intelligence.

[B23-jintelligence-13-00077] Li Chenyu, Ren Xuezhu, Schweizer Karl, Wang Tengfei (2022). Strategy use moderates the relation between working memory capacity and fluid intelligence: A combined approach. Intelligence.

[B24-jintelligence-13-00077] Liu Yaohui, Zhan Peida, Fu Yanbin, Chen Qipeng, Man Kaiwen, Luo Yikun (2023). Using a multi-strategy eye-tracking psychometric model to measure intelligence and identify cognitive strategy in Raven’s advanced progressive matrices. Intelligence.

[B25-jintelligence-13-00077] Loesche Patrick, Wiley Jennifer, Hasselhorn Marcus (2015). How knowing the rules affects solving the Raven Advanced Progressive Matrices Test. Intelligence.

[B26-jintelligence-13-00077] Lozano José H. (2015). Are impulsivity and intelligence truly related constructs? Evidence based on the fixed-links model. Personality and Individual Differences.

[B27-jintelligence-13-00077] Lozano José H., Revuelta Javier (2020). Investigating operation-specific learning effects in the Raven’s Advanced Progressive Matrices: A linear logistic test modeling approach. Intelligence.

[B28-jintelligence-13-00077] Markon Kristian. E. (2019). Bifactor and hierarchical models: Specification, inference, and interpretation. Annual Review of Clinical Psychology.

[B29-jintelligence-13-00077] Marshalek Brachia, Lohman David F., Snow Richard E. (1983). The complexity continuum in the radex and hierarchical models of intelligence. Intelligence.

[B30-jintelligence-13-00077] Martarelli Corinna S., Mast Fred W. (2013). Eye movements during long-term pictorial recall. Psychological Research.

[B31-jintelligence-13-00077] Nagy Gabriel, Ulitzsch Esther, Lindner Marlit A. (2023). The role of rapid guessing and test-taking persistence in modelling test-taking engagement. Journal of Computer Assisted Learning.

[B32-jintelligence-13-00077] Peirce Jonathan, Gray Jeremy R., Simpson Sol, MacAskill Michael, Höchenberger Richard, Sogo Hiroyuki, Kastman Erik, Lindeløv Jonas Kristoffer (2019). PsychoPy2: Experiments in behavior made easy. Behavior Research Methods.

[B33-jintelligence-13-00077] Perret Patrick, Dauvier Bruno (2018). Children’s allocation of study time during the solution of Raven’s progressive matrices. Journal of Intelligence.

[B34-jintelligence-13-00077] Raden Megan J., Jarosz Andrew F. (2022). Strategy Transfer on Fluid Reasoning Tasks. Intelligence.

[B35-jintelligence-13-00077] Raven John (2000). The Raven’s progressive matrices: Change and stability over culture and time. Cognitive Psychology.

[B36-jintelligence-13-00077] Raven John C., Raven John, Court John H. (1998). Advanced Progressive Matrices [Measurement Instrument]. https://www.testzentrale.ch/shop/advanced-progressive-matrices.html.

[B37-jintelligence-13-00077] Ren Xuezhu, Schweizer Karl, Wang Tengfei, Chu Pei, Gong Qin (2017a). On the relationship between executive functions of working memory and components derived from fluid intelligence measures. Acta Psychologica.

[B38-jintelligence-13-00077] Ren Xuezhu, Gong Qin, Chu Pei, Wang Tengfei (2017b). Impulsivity is not related to the ability and position components of intelligence: A comment on Lozano 2015. Personality and Individual Differences.

[B39-jintelligence-13-00077] Ren Xuezhu, Wang Tengfei, Altmeyer Michael, Schweizer Karl (2014). A learning-based account of fluid intelligence from the perspective of the position effect. Learning and Individual Differences.

[B40-jintelligence-13-00077] Revelle William (2015). Package ‘psych’. The Comprehensive R Archive Network.

[B41-jintelligence-13-00077] Rosseel Yves (2012). Lavaan: An R package for structural equation modeling and more. Version 0.5–12 (BETA). Journal of Statistical Software.

[B42-jintelligence-13-00077] Schweizer Karl (2013). A threshold-free approach to the study of the structure of binary data. International Journal of Statistics and Probability.

[B43-jintelligence-13-00077] Schweizer Karl, Troche Stefan (2018). Is the factor observed in investigations on the item-position effect actually the difficulty factor?. Educational and Psychological Measurement.

[B44-jintelligence-13-00077] Schweizer Karl, Schreiner Michael, Gold Andreas (2009). The confirmatory investigation of APM items with loadings as a function of the position and easiness of items: A two-dimensional model of APM. Psychology Science Quarterly.

[B45-jintelligence-13-00077] Schweizer Karl, Reiss Siegbert, Schreiner Michael, Altmeyer Michael (2012). Validity improvement in two reasoning measures following the elimination of the position effect. Journal of Individual Differences.

[B46-jintelligence-13-00077] Schweizer Karl, Troche Stefan, Rammsayer Thomas, Zeller Florian (2021). Inductive reasoning and its underlying structure: Support for difficulty and item position effects. Advances in Cognitive Psychology.

[B47-jintelligence-13-00077] Schweizer Karl, Ren Xuezhu, Wang Tengfei, Millsap Roger, Bolt Daniel, van der Ark L. Andries, Wang Wen-Chung (2015). A Comparison of Confirmatory Factor Analysis of Binary Data on the Basis of Tetrachoric Correlations and of Probability-Based Covariances: A Simulation Study. Quantitative Psychology Research.

[B48-jintelligence-13-00077] Snow Richards E., Lesgold Alan M., Pellegrino James W., Fokkema Sipke D., Glaser Robert (1978). Eye Fixation and Strategy Analyses of Individual Differences in Cognitive Aptitudes. Cognitive Psychology and Instruction.

[B49-jintelligence-13-00077] Snow Richard E., Snow Richard E., Federico Pat-Anthony, Montague William E. (1980). Aptitude Processes. Aptitude, Learning, and Instruction: Cognitive Process Analyses of Aptitude.

[B50-jintelligence-13-00077] Spearman Charles (1927). The Nature of ‘‘Intelligence’’ and the Principles of Cognition.

[B51-jintelligence-13-00077] SR Research (2016). Eyelink 1000 Plus [Apparatus and Software]. https://www.sr-research.com/eyelink-1000-plus/.

[B52-jintelligence-13-00077] Verguts Tom, Boeck Paul De (2002). The induction of solution rules in Raven’s Progressive Matrices Test. European Journal of Cognitive Psychology.

[B53-jintelligence-13-00077] Vigneau François, Caissie André. F., Bors Douglas A. (2006). Eye-movement analysis demonstrates strategic influences on intelligence. Intelligence.

[B54-jintelligence-13-00077] von Gugelberg Helene M., Schweizer Karl, Troche Stefan J. (2021). The dual mechanisms of cognitive control and their relation to reasoning and the item-position effect. Acta Psychologica.

[B55-jintelligence-13-00077] von Gugelberg Helene M., Schweizer Karl, Troche Stefan J. (2025). Experimental evidence for rule learning as the underlying source of the item-position effect in reasoning ability measures. Learning and Individual Differences.

[B56-jintelligence-13-00077] Zeller Florian, Reiss Siegbert, Schweizer Karl (2017). Is the item-position effect in achievement measures induced by increasing item difficulty?. Structural Equation Modeling: A Multidisciplinary Journal.

