# Supplementary material for: Individual Differences in Strategy and the Item-Position Effect in Reasoning Ability Measures
Source: J Intell. 2025 Jun 26;13(7):77. doi: 10.3390/jintelligence13070077 (PMC12295732; doi:10.3390/jintelligence13070077)
Supplement: Supplementary file 1 [file jintelligence-13-00077-s001.zip › Full_sub.pdf]

Supplementary materials

Individual Differences in Strategy and the Item-Position Effect in Reasoning Ability Measures

Given are all Figures and Tables presented in the manuscript, yet with different cut-offs. Figures always depict values for all 36 items to allow for visual comparisons of the different subsamples regarding their performance.

Cut-off at item 22, includes all participants (N = 210) who completed the first 22 items of the Raven Progressive Matrices. Model estimation was based on the 22 first items and the 210 participants who completed these items.

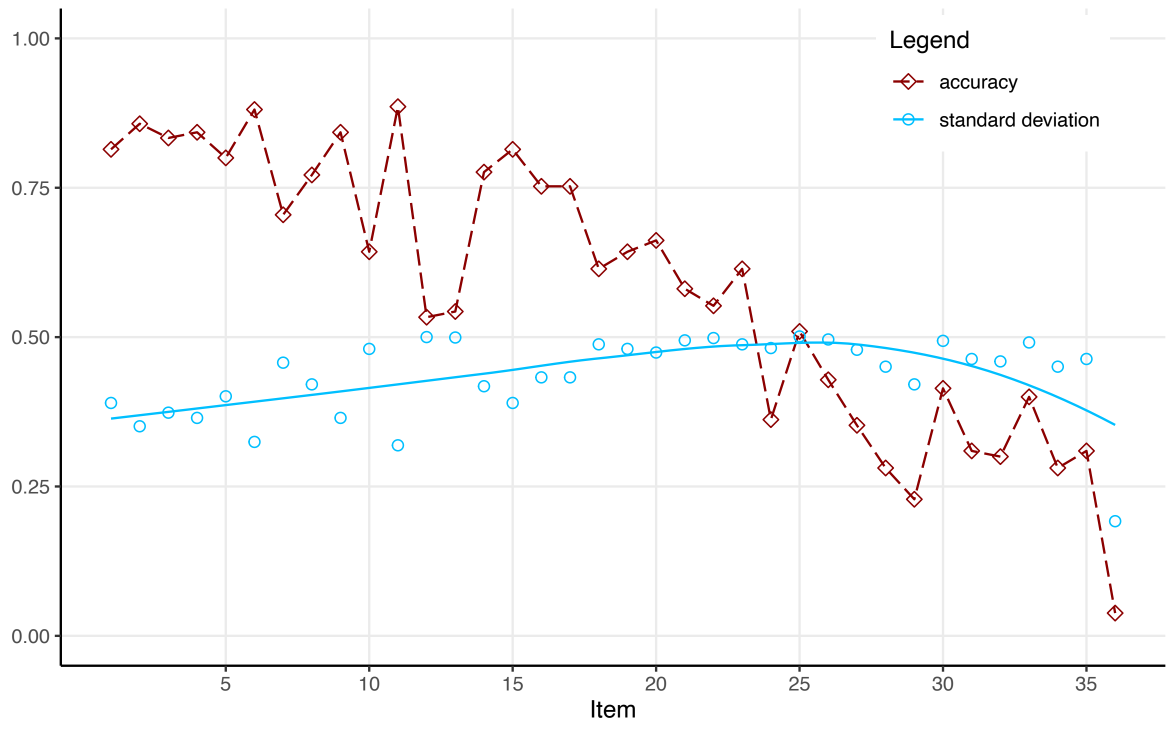

Figure S3_22. Difficulty (P_i) as mean of correct responses and standard deviation for all APM items. Difficulty is depicted by the dashed line and little squares. Little circles and the solid polynomial regression depict standard deviation. Values calculated for all 210 participants, across all 36 items

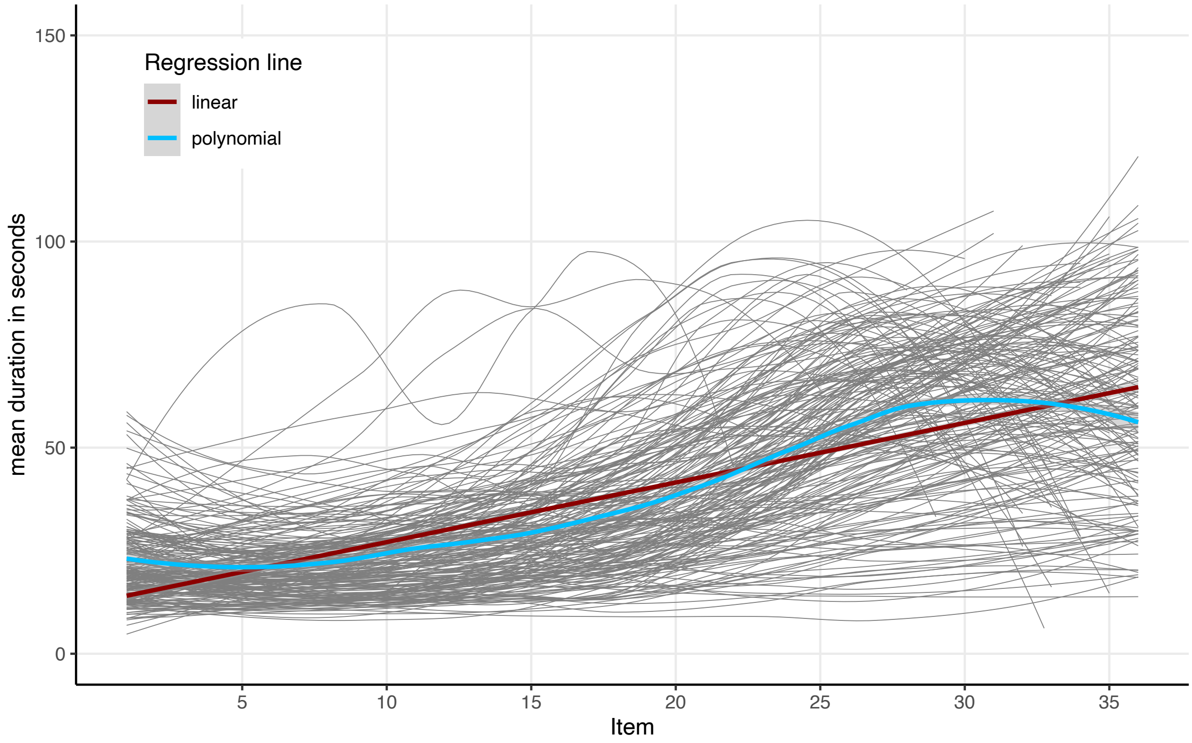

Figure S4_22. Mean item latency for all 36 items of the 210 participants who completed at least the first 22 items (gray), linear (red) and local polynomial (blue) regression fitted line for analyzed sample.

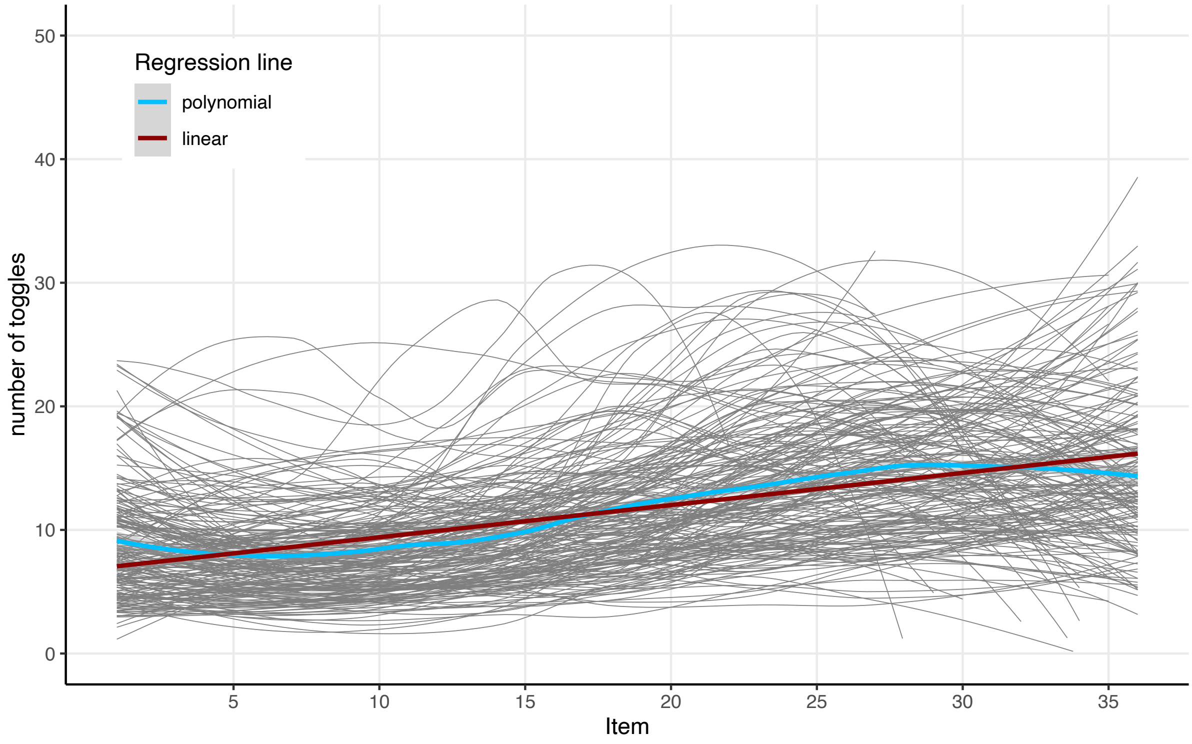

Figure S5_22. Absolute number of toggles for all 36 items of the 210 participants who completed at least the first 22 items (gray), linear (red) and local polynomial (blue) regression fitted line for analyzed sample.

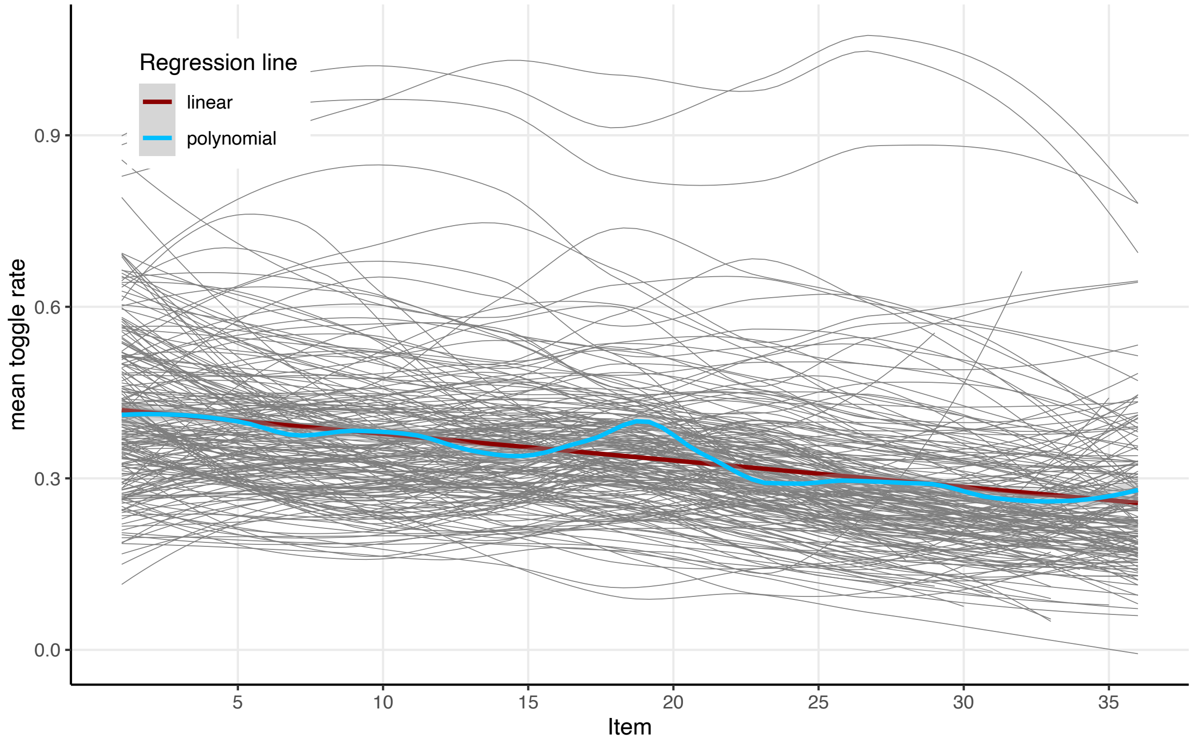

Figure S6_22. Toggle rate for all 36 items of the 210 participants who completed at least the first 22 items (gray), linear (red) and local polynomial (blue) regression fitted line for analyzed sample.

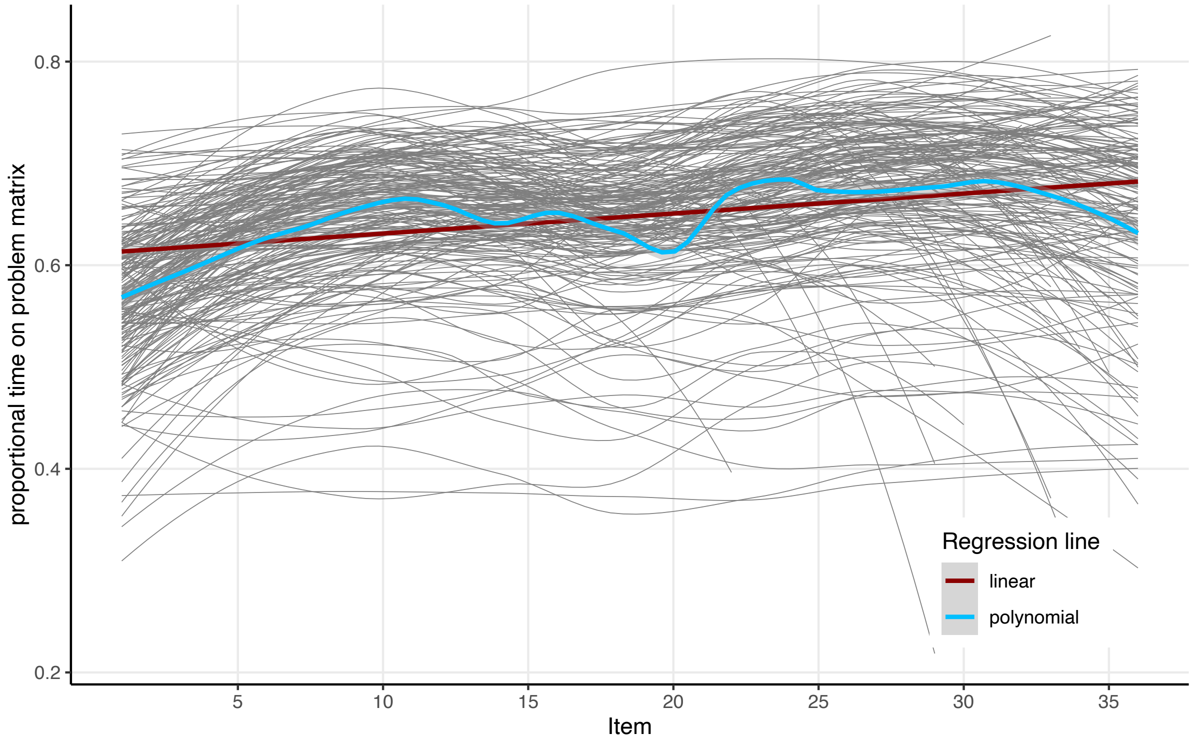

Figure S7_22. Proportional time on problem matrix for all 36 items of the 210 participants who completed at least the first 22 items (gray), linear (red) and local polynomial (blue) regression fitted line for analyzed sample.

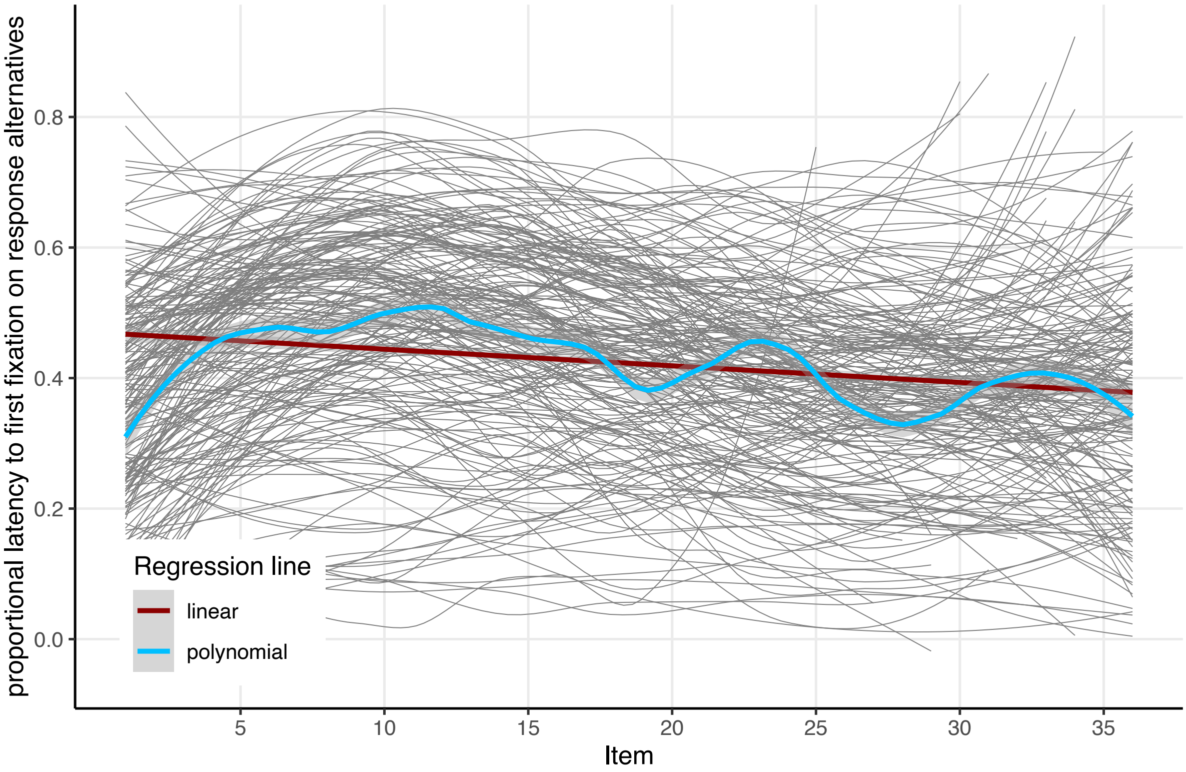

Figure S8_22. Proportional time to first fixation on response alternatives for all 36 items of the 210 participants who completed at least the first 22 items (gray), linear (red) and local polynomial (blue) regression fitted line for analyzed sample.

Table S1_22. Correlation matrix of APM score, eye tracking metrics and strategy questionnaire for participants completing 22 items on the APM

	APM	Tog Rate	N of Tog	I-Latency	T on M	T on RA	FF on RA	Pr. T on M	Pr. T on RA	Pr. T to FF
APM Score	APM	0.000	0.010	0.009	0.000	0.553	0.000	0.000	0.000	0.000
Toggle Rate	-0.578	Tog Rate	0.000	0.000	0.000	0.350	0.000	0.000	0.000	0.000
Number of Toggles	-0.177	0.352	N of Tog	0.000	0.000	0.000	0.113	0.005	0.003	0.000
Item Latency	0.180	-0.298	0.741	I-Latency	0.000	0.000	0.000	0.001	0.000	0.000
Time on Matrix	0.273	-0.401	0.634	0.973	T on M	0.000	0.000	0.000	0.000	0.002
Time on Response Alternatives	*-0.041*	*0.065*	0.855	0.802	0.707	T on RA	0.394	0.211	0.000	0.000
Latency to First Fixation on RA	0.402	-0.713	*-0.110*	0.475	0.570	*0.059*	FF on RA	0.000	0.000	0.000
Proportional Time on Matrix	0.545	-0.655	-0.193	0.223	0.416	*-0.087*	0.529	Pr. T on M	0.000	0.000
Proportional Time on Response Alternatives	-0.434	0.708	0.202	-0.274	-0.373	0.271	-0.611	-0.603	Pr. T on RA	0.000
Proportional Time to First Fixation on RA	0.440	-0.629	-0.745	-0.337	-0.215	-0.585	0.561	0.467	-0.496	Pr. T to FF

Note. Given are correlations in the lower triangular matrix (non- significant values are *italic*), with the corresponding *p*-value in the upper triangular matrix. Abbreviations in the first row and diagonally across the matrix correspond to information given in the first column. APM Score was calculated as the number of correct answers given within 22 items. All values were calculated for all 22 items and the 210 participants who completed the items.

Table S2_22. Goodness-of-fit indices and criteria for each of the calculated measurement models.

	χ^2 (*df*)	*p*	CFI	RMSEA	SRMR	AIC
APM scores						
one-factor model / Model A	266.15 (209)	0.005	0.926	0.039	0.058	4312
**bifactor model / Model B**	**257.56 (208)**	**0.011**	**0.936**	**0.036**	**0.057**	**4302**
bifactor model / Model C	258.63. (208)	0.010	0.935	0.037	0.057	4304
Toggle rate						
one-factor model / Model A	320.23 (209)	<.001	0.922	0.053	0.050	-4111
**bifactor model / Model B**	**291.80 (208)**	**<.001**	**0.941**	**0.046**	**0.049**	**-4140**
bifactor model / Model C	298.23 (208)	<.001	0.937	0.048	0.048	-4134
Proportional Time on Matrix						
one-factor model / Model A	292.34 (209)	<.001	0.951	0.045	0..045	-10269
**bifactor model / Model B**	**277.83 (208)**	**0.001**	**0.960**	**0.041**	**0.045**	**-10285**
bifactor model / Model C	278.90 (208)	0.001	0.959	0.042	0.044	-10284
Proportional time to first fixation on response alternatives						
one-factor model / Model A	264.75 (209)	<.001	0.948	0.037	0.053	-709
**bifactor model / Model B**	**240.47 (208)**	**0.005**	**0.970**	**0.029**	**0.051**	**-735**
bifactor model / Model C	248.08 (208)	0.002	0.963	0.032	0.051	-726
Full Models with Model B for the APM score data and the best fitting model for the respective eye tracking metric						
Toggle rate / Model B	1155.59 (896)	<.001	0.889	0.039	0.062	73
Proportional time on Matrix / Model B	1206.51 (896)	<.001	0.886	0.041	0.064	-6054
Proportional time to first fixation on response alternatives / Model B	1183.96 (896)	<.001	0.868	0.040	0.064	3506

Note. Model A is the one-factor model. Model B includes a second latent variable with linearly, and Model C with quadratically increasing factor loadings from the first to the last item. Models in **bold** indicate the best fitting model.

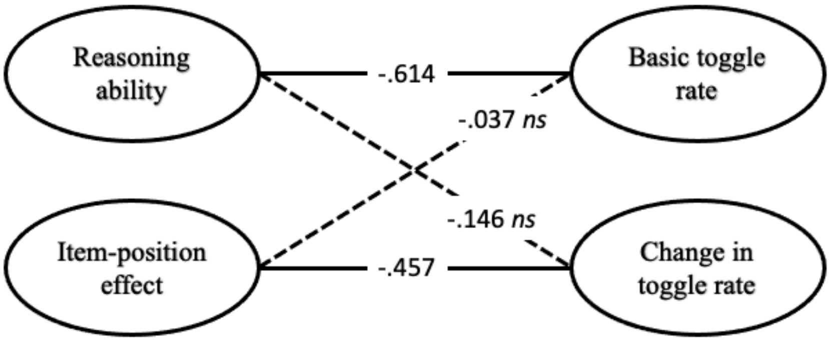

Figure S9_22. Correlations between latent variables of the final model regarding toggle rate.

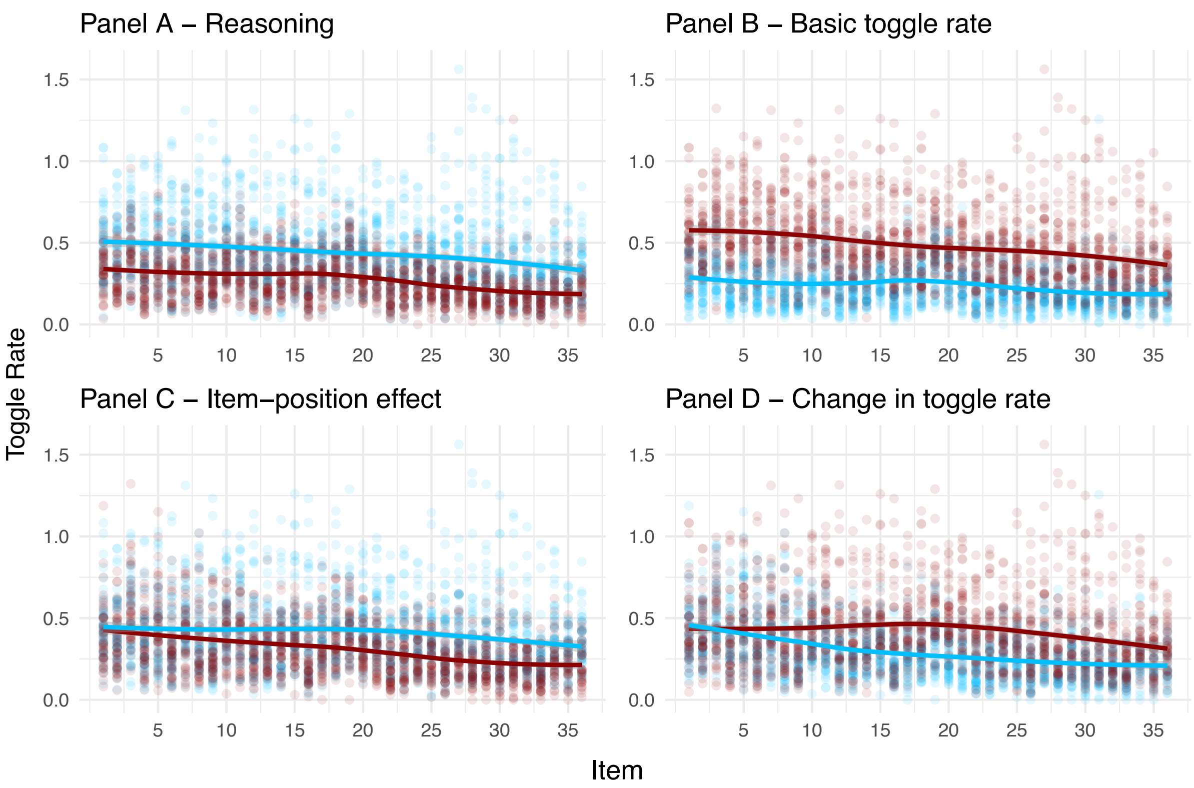

Figure S10_22. Toggle rate for participants with high or low values on the latent variables. *Note.* Blue lines represent participants with low factor scores on the latent variable. Red lines represent participants with high factor scores on the depicted latent variable.

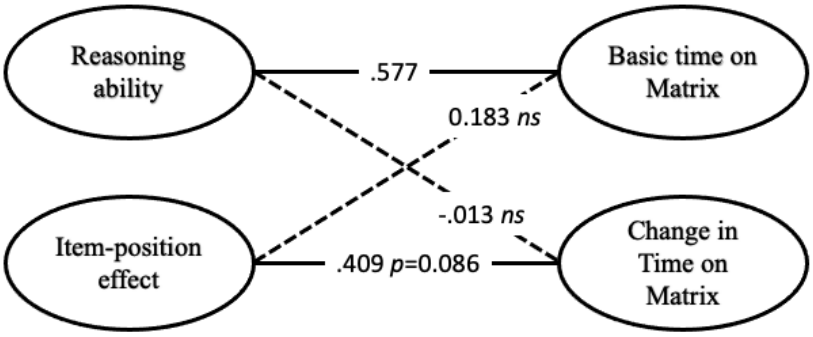

Figure S11_22. Correlations between latent variables of the full model regarding the proportional time on the problem matrix.

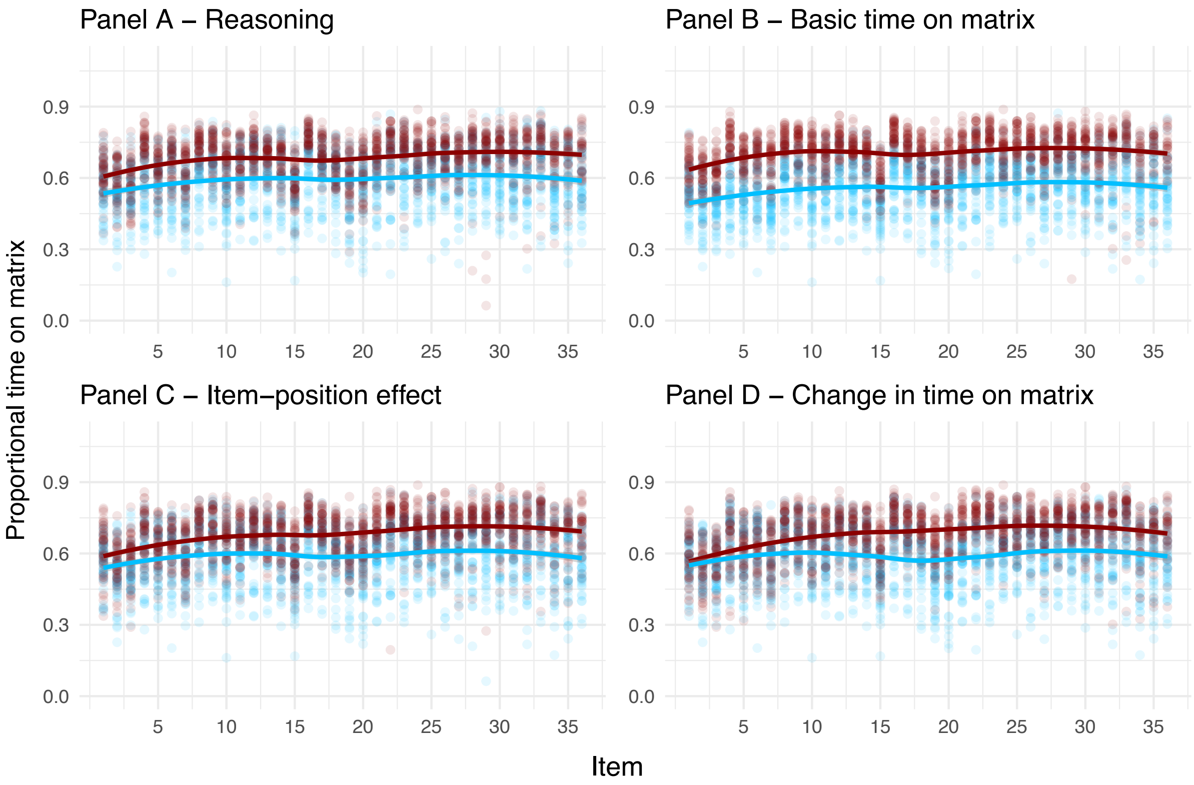

Figure S12_22. Proportional time on matrix for participants with high or low values on the latent variables. *Note.* Blue lines represent participants with low factor scores on the latent variable. Red lines represent participants with high factor scores on the depicted latent variable.

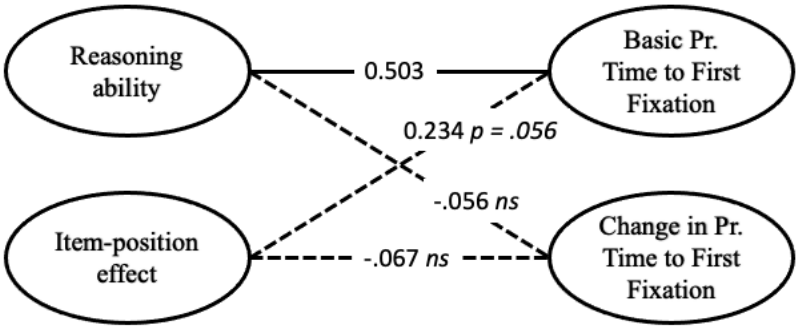

Figure S13_22. Correlations between latent variables of the full model regarding the proportional time on the problem matrix. Solid lines indicate significant correlations, dashed lines non-significant ones.

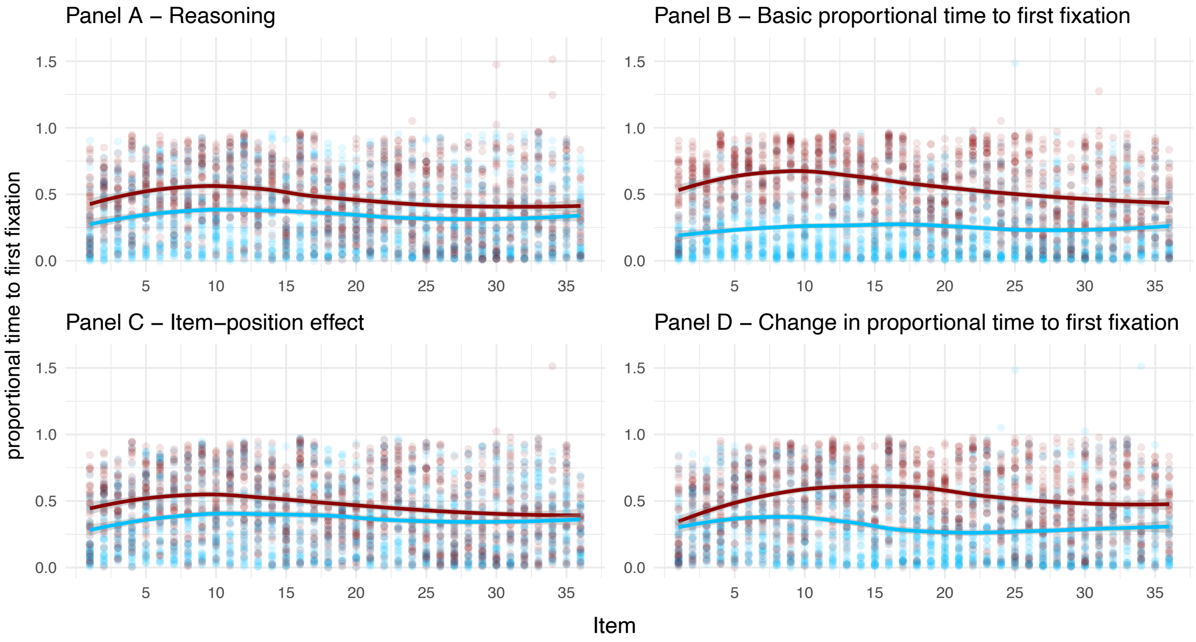

Figure S14_22. Proportional time to first fixation on response alternatives for participants with high or low values on the latent variables. *Note.* Blue lines represent participants with low factor scores on the latent variable. Red lines represent participants with high factor scores on the depicted latent variable.

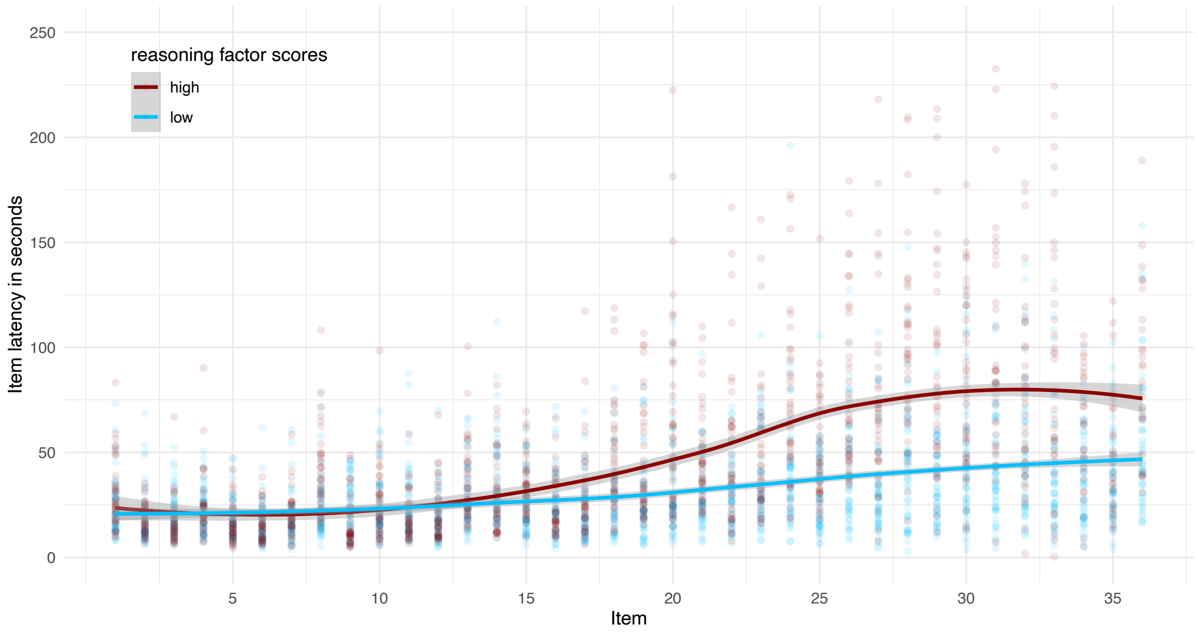

Figure S15_22. Item Latencies for participants with high or low reasoning ability factor scores. *Note*. Given are item latencies in seconds and a polynomial trend line for the participants with the 50 highest (in red) and 50 lowest (in blue) reasoning ability factor scores of the final model.

Cut-off at item 27, includes all participants (n = 206) who completed the first 27 items of the Raven Progressive Matrices. Model estimation was based on the 27 first items and the 206 participants who completed these items.

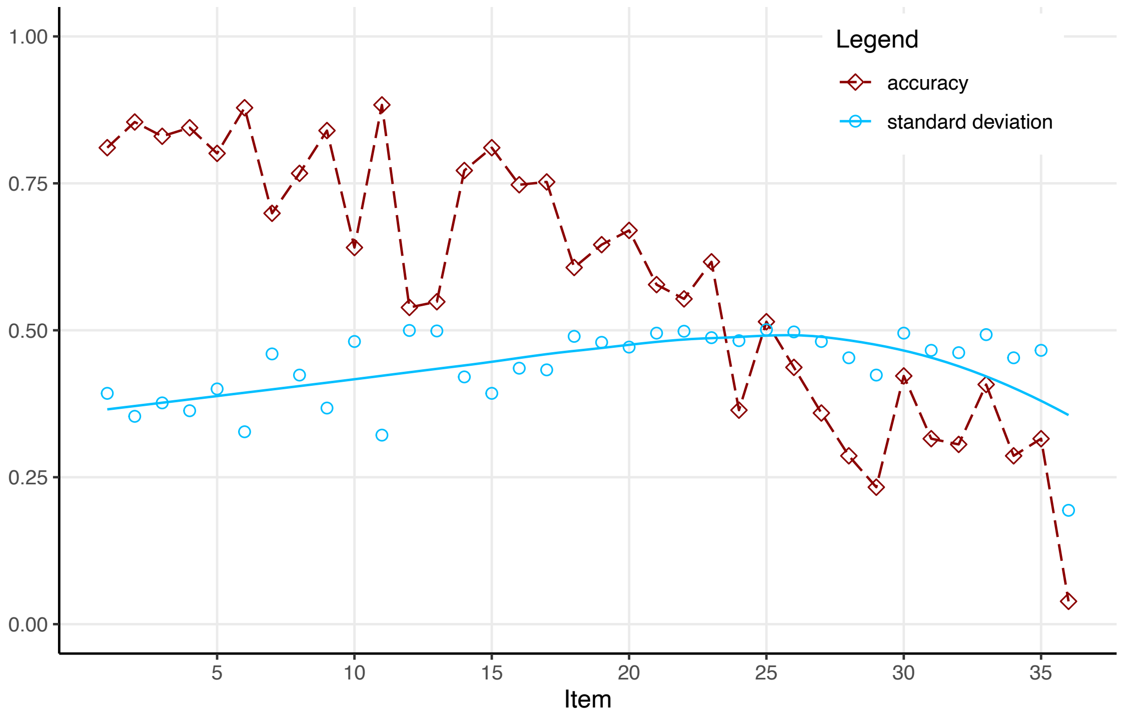

Figure S3_27. Difficulty (P_i) as mean of correct responses and standard deviation for all APM items. Difficulty is depicted by the dashed line and little squares. Little circles and the solid polynomial regression depict standard deviation. Values are based on all 36 items of participants who completed at least the first 27 items.

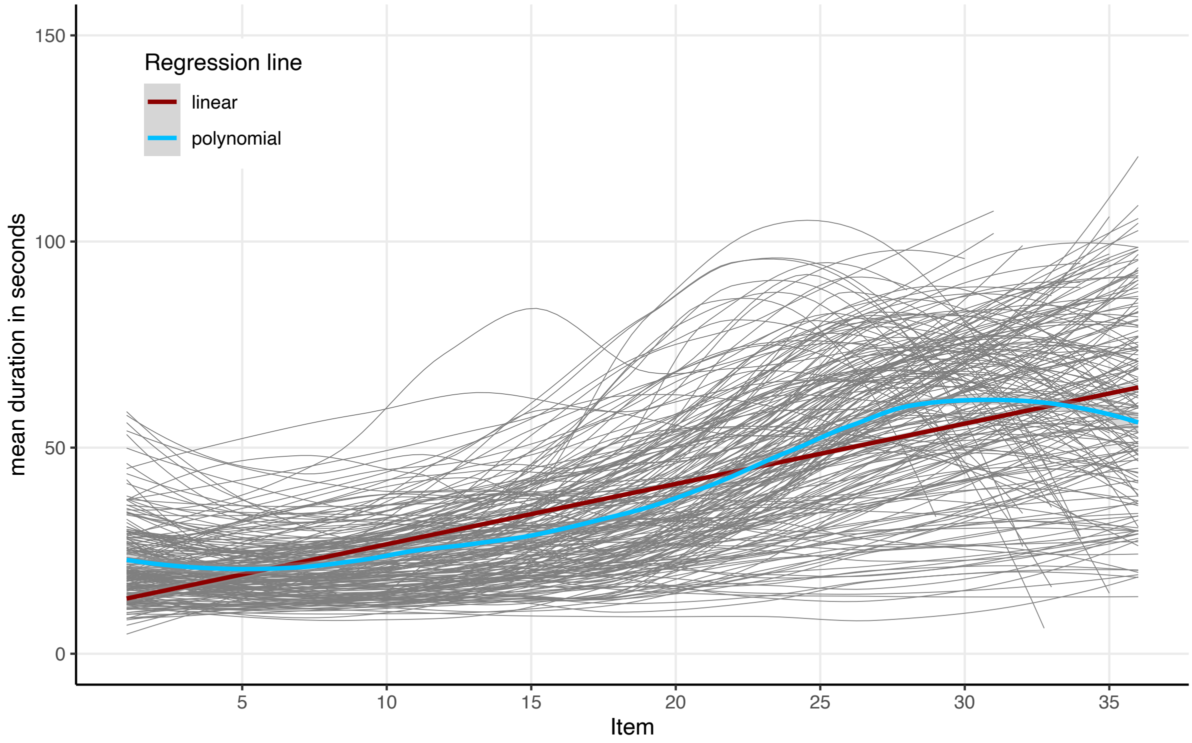

Figure S4_27. Mean item latency for each participant (gray), linear (red) and local polynomial (blue) regression fitted line for analysed sample. Values are based on all 36 items of participants who completed at least the first 27 items.

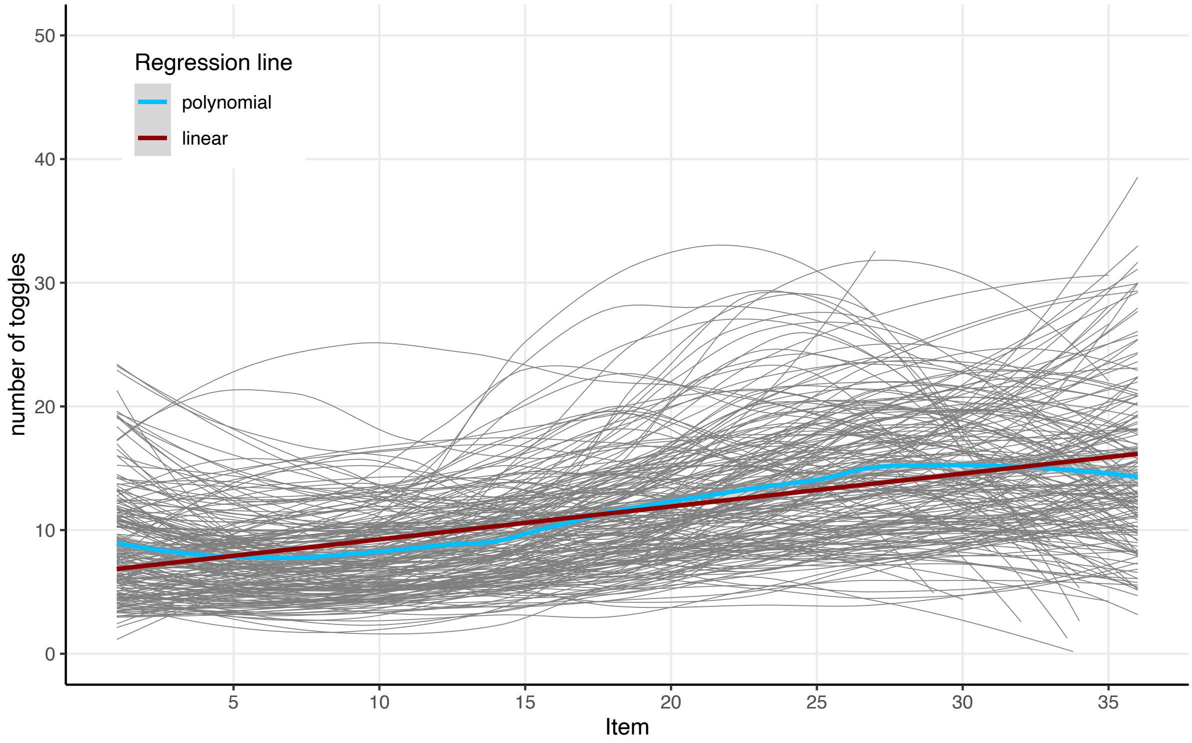

Figure S5_27. Absolute number of toggles for each participant (gray), linear (red) and local polynomial (blue) regression fitted line for analysed sample. Values are based on all 36 items of participants who completed at least the first 27 items.

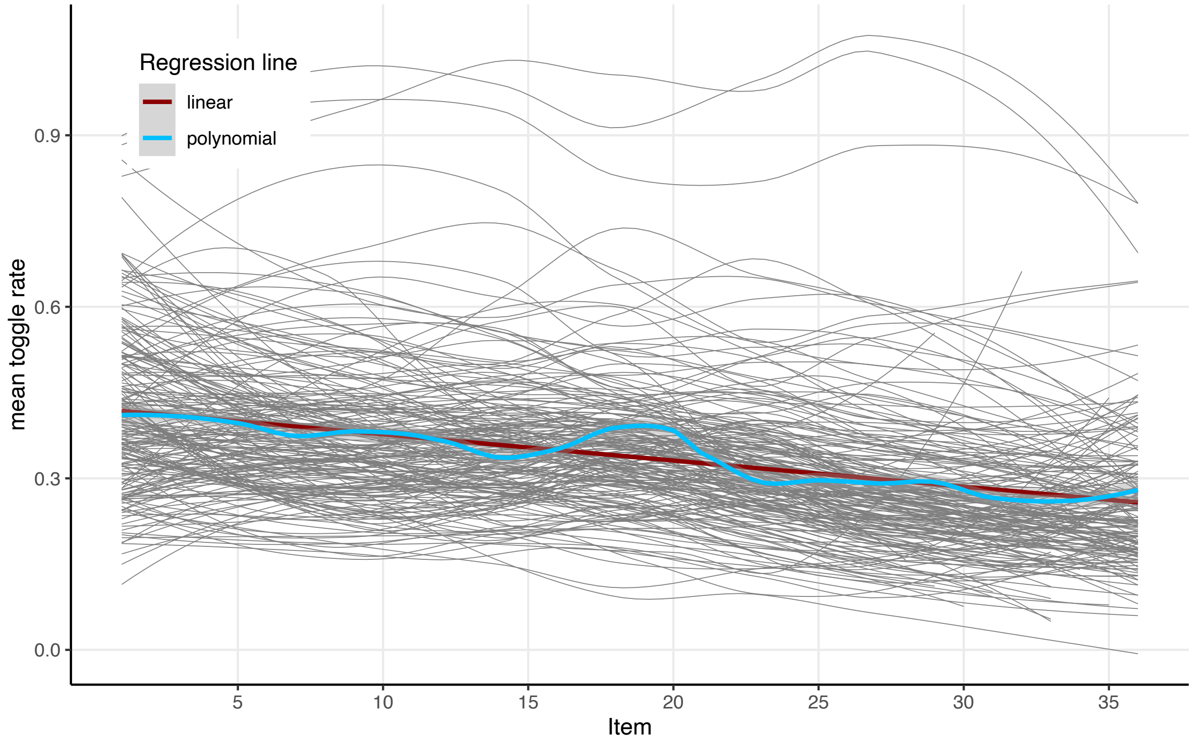

Figure S6_27. Toggle rate for each participant (gray), linear (red) and local polynomial (blue) regression fitted line for analysed sample. Values are based on all 36 items of participants who completed at least the first 27 items.

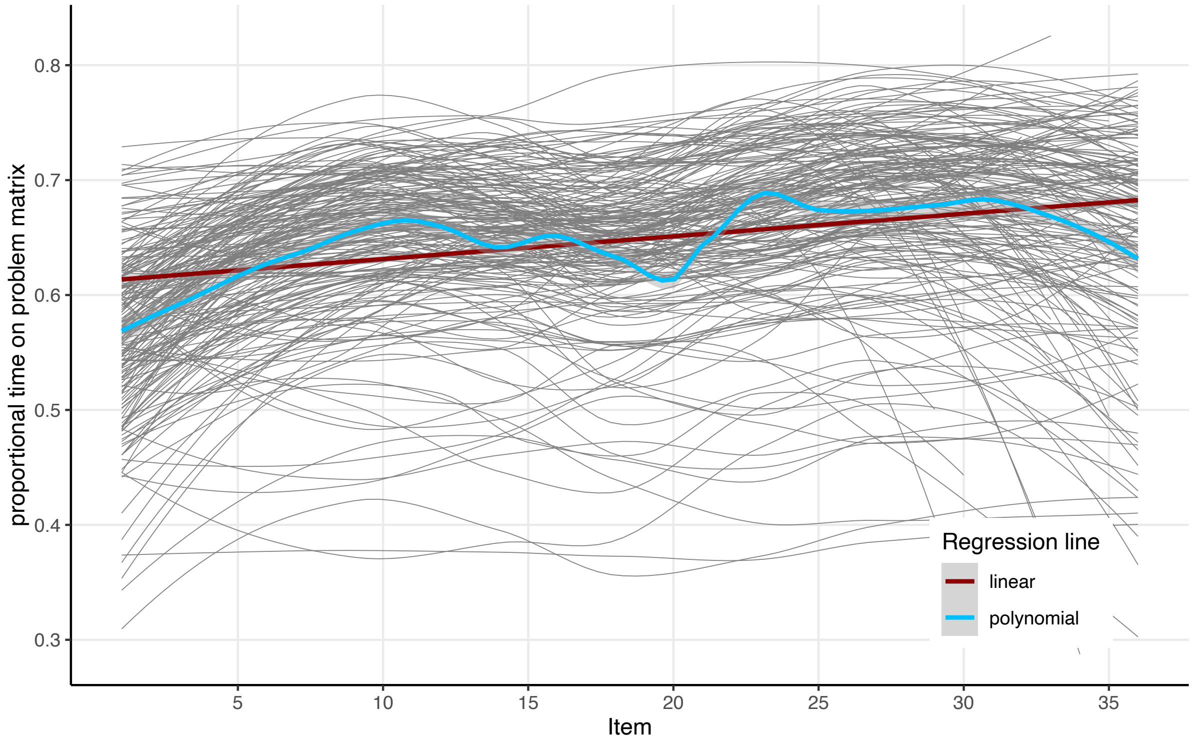

Figure S7_27. Proportional time on problem matrix for each participant (gray), linear (red) and local polynomial (blue) regression fitted line for analysed sample. Values are based on all 36 items of participants who completed at least the first 27 items.

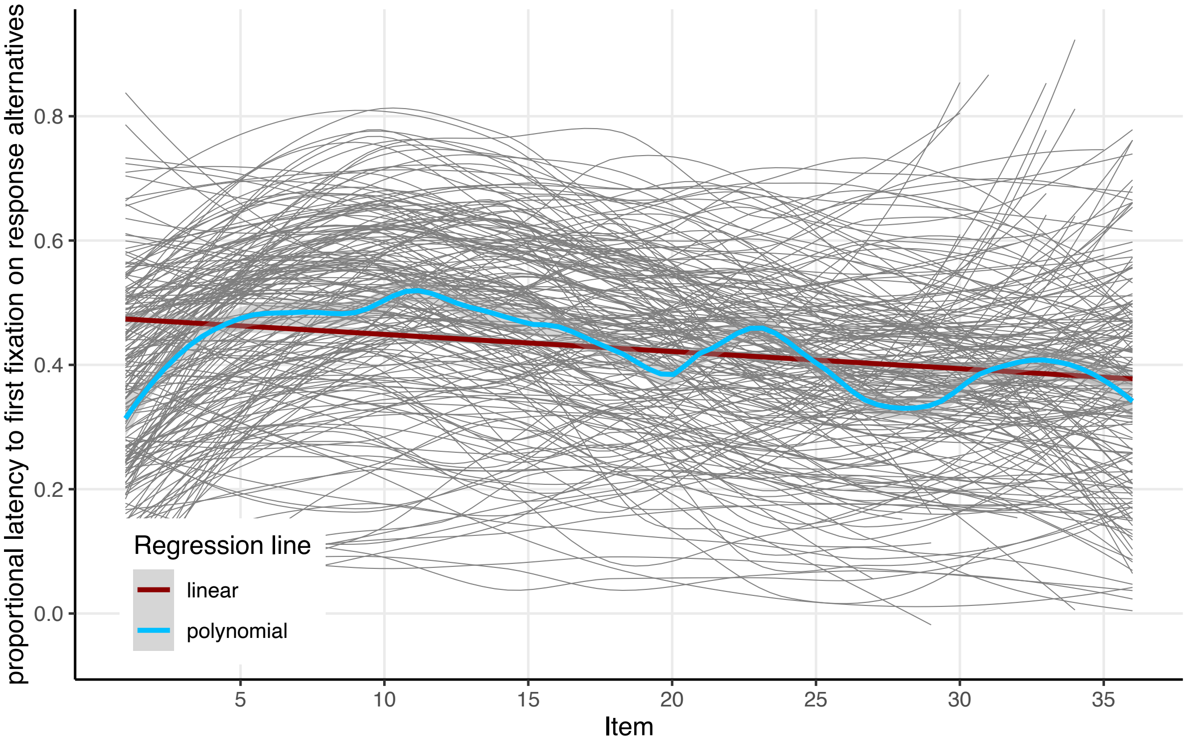

Figure S8_27. Latency to first fixation on response alternatives for each participant (gray), linear (red) and local polynomial (blue) regression fitted line for analysed sample. Values are based on all 36 items of participants who completed at least the first 27 items.

Table S1_27. Correlation matrix of APM score, eye tracking metrics and strategy questionnaire for participants completing 27 items on the APM

	APM	Tog Rate	N of Tog	I-Latency	T on M	T on RA	FF on RA	Pr. T on M	Pr. T on RA	Pr. T to FF
APM - Score	APM	0.000	0.012	0.000	0.000	0.915	0.000	0.000	0.000	0.000
Toggle Rate	-0.611	Tog Rate	0.000	0.000	0.000	0.529	0.000	0.000	0.000	0.000
Number of Toggles	-0.175	0.353	N of Tog	0.000	0.000	0.000	0.190	0.013	0.005	0.000
Item Latency	0.275	-0.389	0.658	I-Latency	0.000	0.000	0.000	0.000	0.000	0.004
Time on Matrix	0.362	-0.477	0.548	0.974	T on M	0.000	0.000	0.000	0.000	0.282
Time on Response Alternatives	*0.007*	*0.044*	0.823	0.749	0.661	T on RA	0.081	0.685	0.000	0.000
Latency to First Fixation on RA	0.456	-0.722	*-0.092*	0.586	0.660	*0.122*	Pr. T on M	0.000	0.000	0.000
Proportional Time on Matrix	0.557	-0.671	-0.172	0.314	0.497	*-0.028*	0.536	Pr. T on RA	0.000	0.000
Proportional Time on Response Alternatives	-0.451	0.746	0.197	-0.366	-0.442	0.259	-0.616	-0.598	FF on RA	0.000
Proportional Time to First Fixation on RA	0.478	-0.649	-0.716	-0.198	*-0.075*	-0.545	0.584	0.477	-0.521	Pr. T to FF

Note. Given are correlations in the lower triangular matrix (non- significant values are *italic*), with the corresponding *p*-value in the upper triangular matrix. Abbreviations in the first row and diagonally across the matrix correspond to information given in the first column. APM Score was calculated as the number of correct answers given within the first 27 items. All values were calculated for the first 27 items and the 206 participants who completed the items.

Table S2_27. Goodness-of-fit indices and criteria for each of the calculated measurement models.

	χ^2 (*df*)	*p*	CFI	RMSEA	SRMR	AIC
APM scores						
one-factor model / Model A	413.13 (324)	0.001	0.908	0.038	0.059	5596
**bifactor model / Model B**	**294.92 (323)**	**0.004**	**0.926**	**0.034**	**0.059**	**5578**
bifactor model / Model C	394.84 (323)	0.004	0.926	0.034	0.058	5579
Toggle rate						
one-factor model / Model A	454.10 (324)	<.001	0.931	0.046	0.047	-5512
**bifactor model / Model B**	**424.44 (323)**	**<.001**	**0.946**	**0.041**	**0.047**	**-5541**
bifactor model / Model C	431.11 (323)	<.001	0.942	0.042	0.046	-5534
Proportional Time on Matrix						
one-factor model / Model A	454.33 (324)	<.001	0.942	0.046	0.045	-12606
bifactor model / Model B	437.62 (323)	0.001	0.949	0.043	0.046	-12624
**bifactor model / Model C**	**435.52 (323)**	**0.001**	**0.950**	**0.043**	**0.045**	**-12626**
Proportional time to first fixation on response alternatives						
one-factor model / Model A	400.12 (324)	0.002	0.938	0.035	0.055	-716
**bifactor model / Model B**	**370.34 (323)**	**0.036**	**0.962**	**0.028**	**0.054**	**-745**
bifactor model / Model C	378.44 (323)	0.018	0.955	0.030	0.054	-736
Full Models with Model B for the APM score data and the best fitting model for the respective eye tracking metric						
Toggle rate / Model B	1666.78 (1371)	<.001	0.900	0.033	0.060	-71
Proportional time on Matrix / Model C	1818.42 (1371)	<.001	0.873	0.041	0.063	-7125
Proportional time to first fixation on response alternatives / Model B	1805.99 (1371)	<.001	0.835	0.040	0.066	4718

Note. Model A is the one-factor model. Model B includes a second latent variable with linearly, and Model C with quadratically increasing factor loadings from the first to the last item. Models in **bold** indicate the best fitting model.

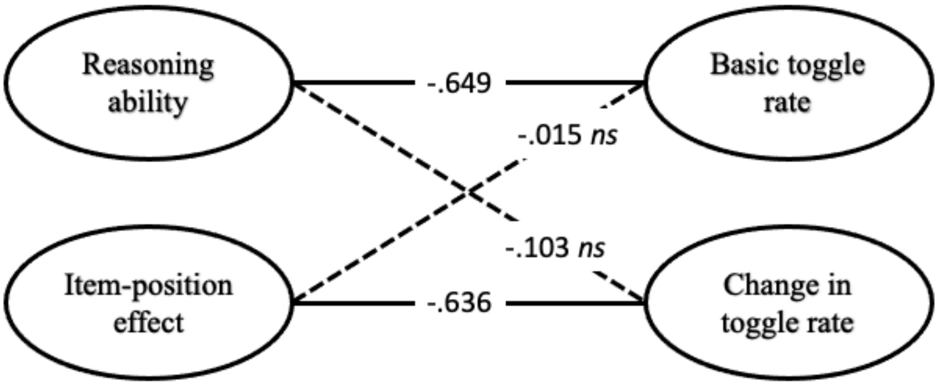

Figure S9_27. Correlations between latent variables of the final model regarding toggle rate for participants who completed the first 27 items.

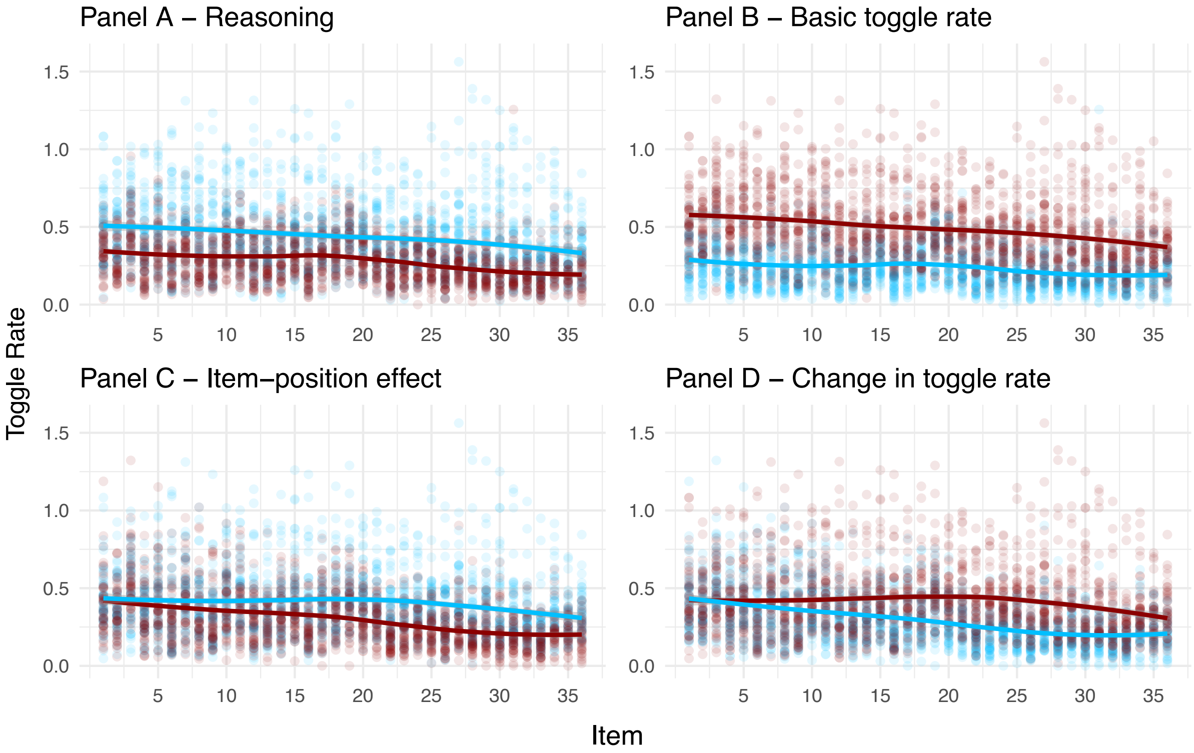

Figure S10_27. Toggle rate for participants with high or low values on the latent variables. *Note.* Blue lines represent participants with low factor scores on the latent variable. Red lines represent participants with high factor scores on the depicted latent variable.

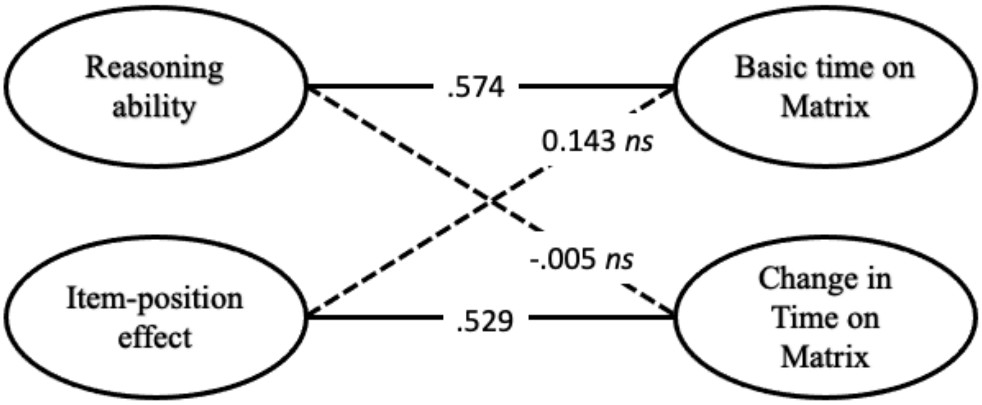

Figure S11_27. Correlations between latent variables of the final model regarding the proportional time on the problem matrix for participants who completed the first 27 items.

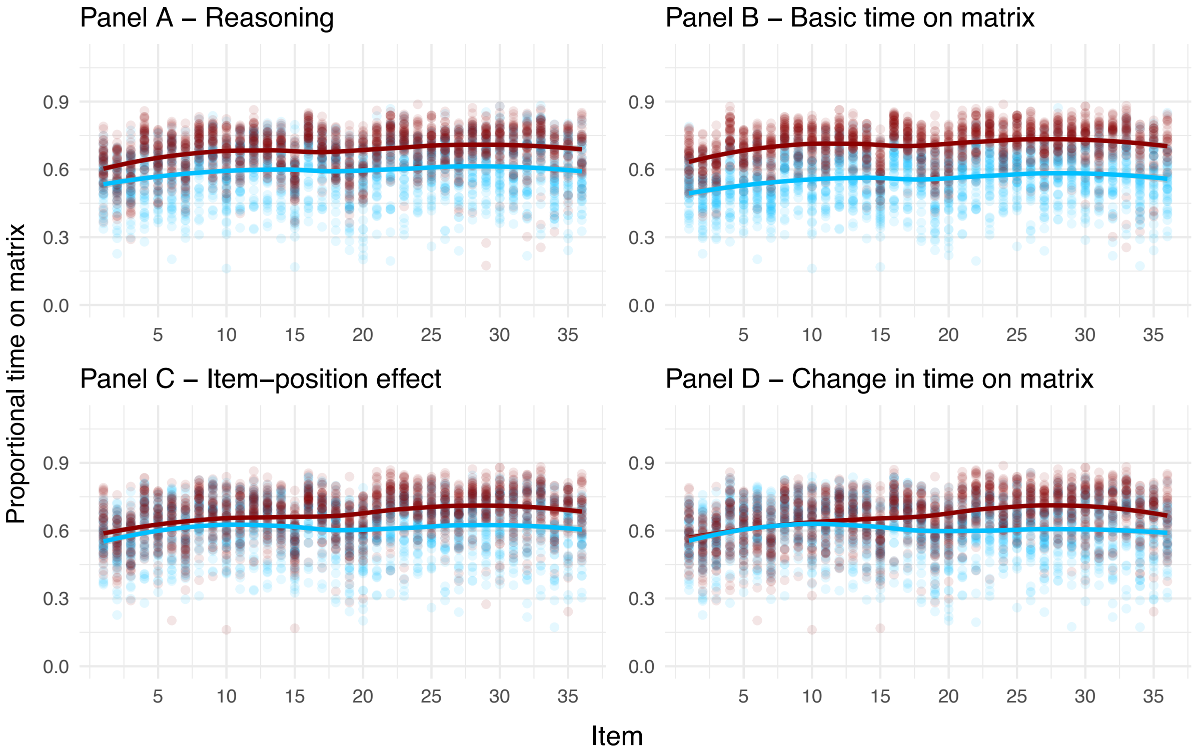

Figure S12_27. Proportional time on matrix for participants with high or low values on the latent variables. *Note.* Blue lines represent participants with low factor scores on the latent variable. Red lines represent participants with high factor scores on the depicted latent variable.

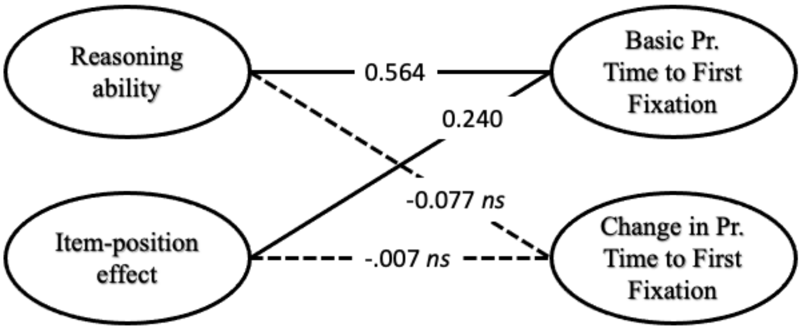

Figure S13_27. Correlations between latent variables of the final model regarding the proportional time to first fixation on response alternatives for participants who completed the first 27 items.

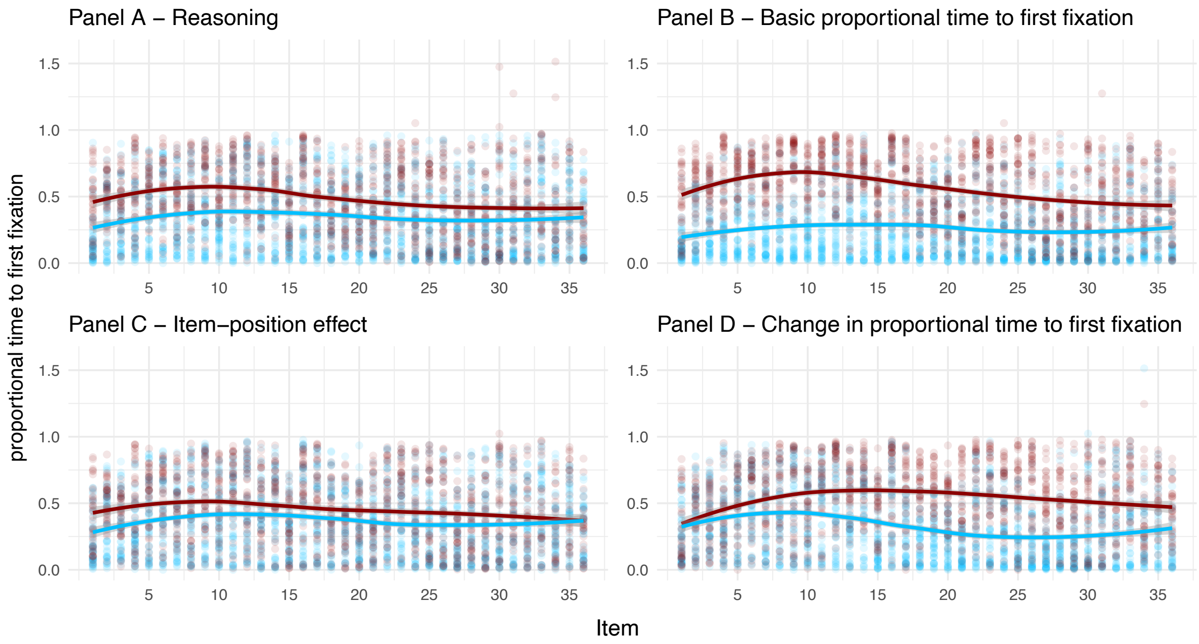

Figure S14_27. Proportional time to first fixation on response alternatives for participants with high or low values on the latent variables. *Note.* Blue lines represent participants with low factor scores on the latent variable. Red lines represent participants with high factor scores on the depicted latent variable.

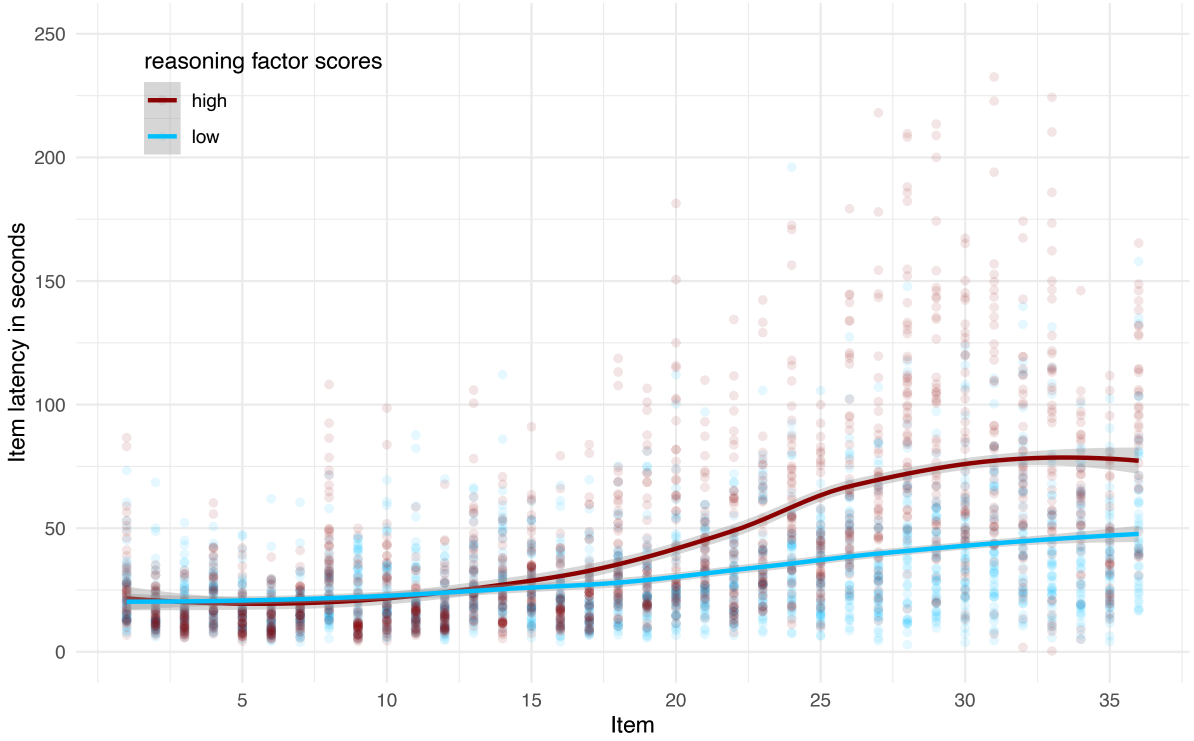

Figure S15_27. Item Latencies for participants with high or low reasoning ability factor scores. Note. Given are item latencies in seconds and a polynomial trend line for the participants with the 50 highest (in red) and 50 lowest (in blue) reasoning ability factor scores of the final model.

Cut-off at item 33, includes all participants (n = 185) who completed the first 27 items of the Raven Progressive Matrices. Model estimation was based on the 27 first items and the 185 participants who completed these items.

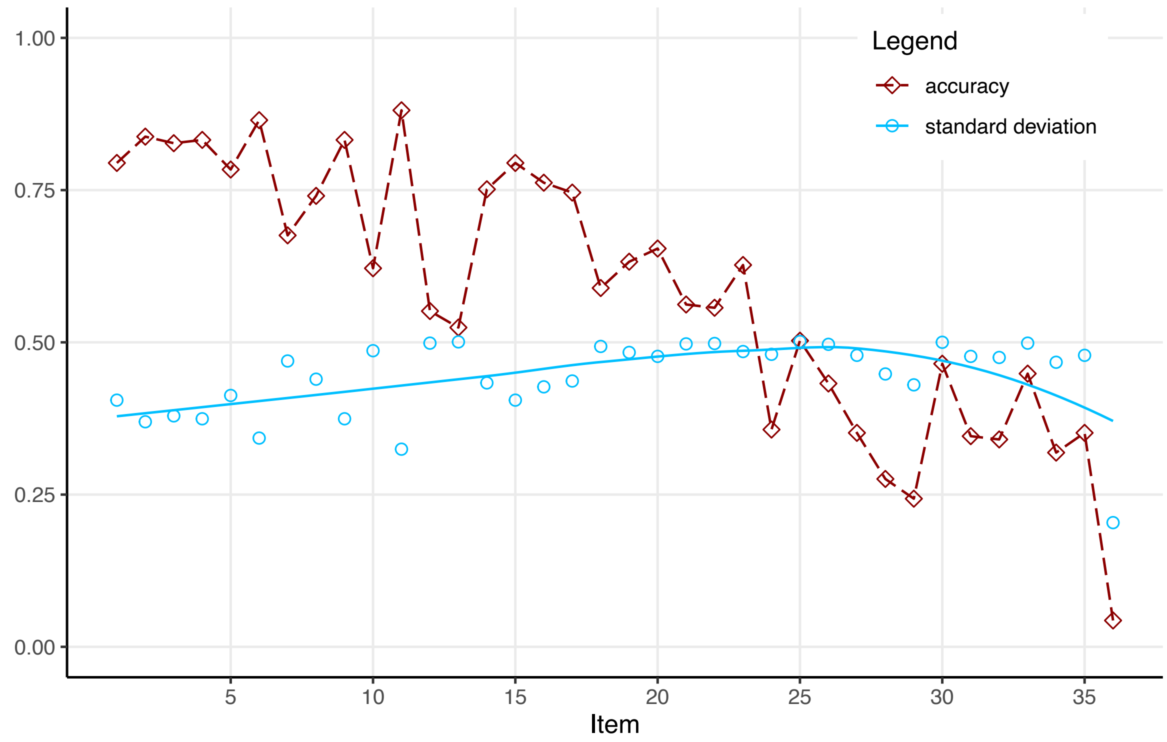

Figure S3_33. Difficulty (P_i) as mean of correct responses and standard deviation for all APM items. Difficulty is depicted by the dashed line and little squares. Little circles and the solid polynomial regression depict standard deviation. Values are based on all 36 items of participants who completed at least the first 33 items.

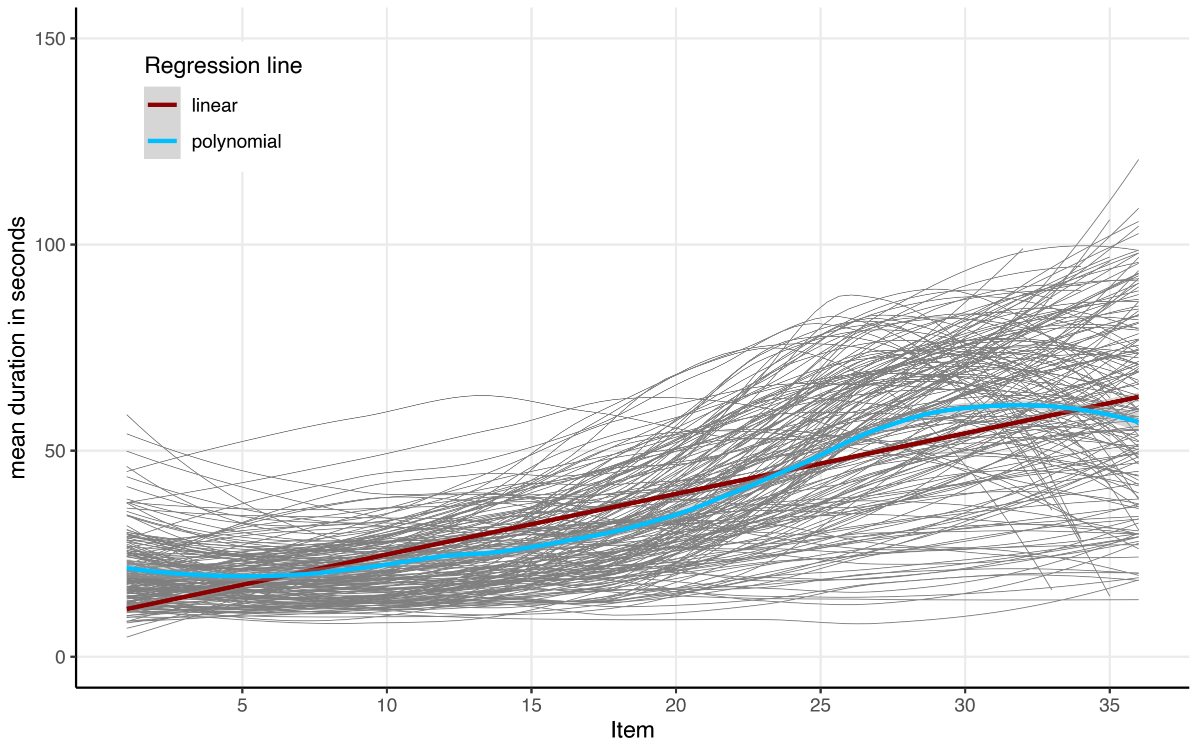

Figure S4_33. Mean item latency for each participant (gray), linear (red) and local polynomial (blue) regression fitted line for analysed sample. Values are based on all 36 items of participants who completed at least the first 33 items.

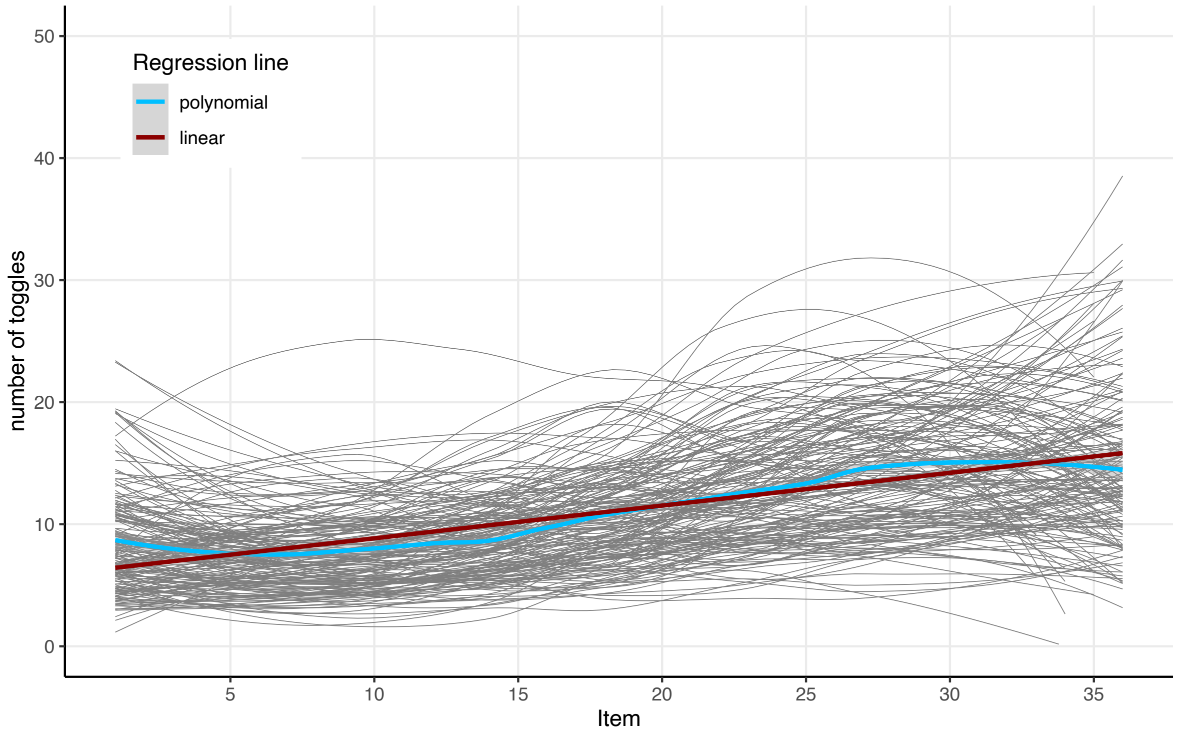

Figure S5_33. Absolute number of toggles for each participant (gray), linear (red) and local polynomial (blue) regression fitted line for analysed sample. Values are based on all 36 items of participants who completed at least the first 33 items.

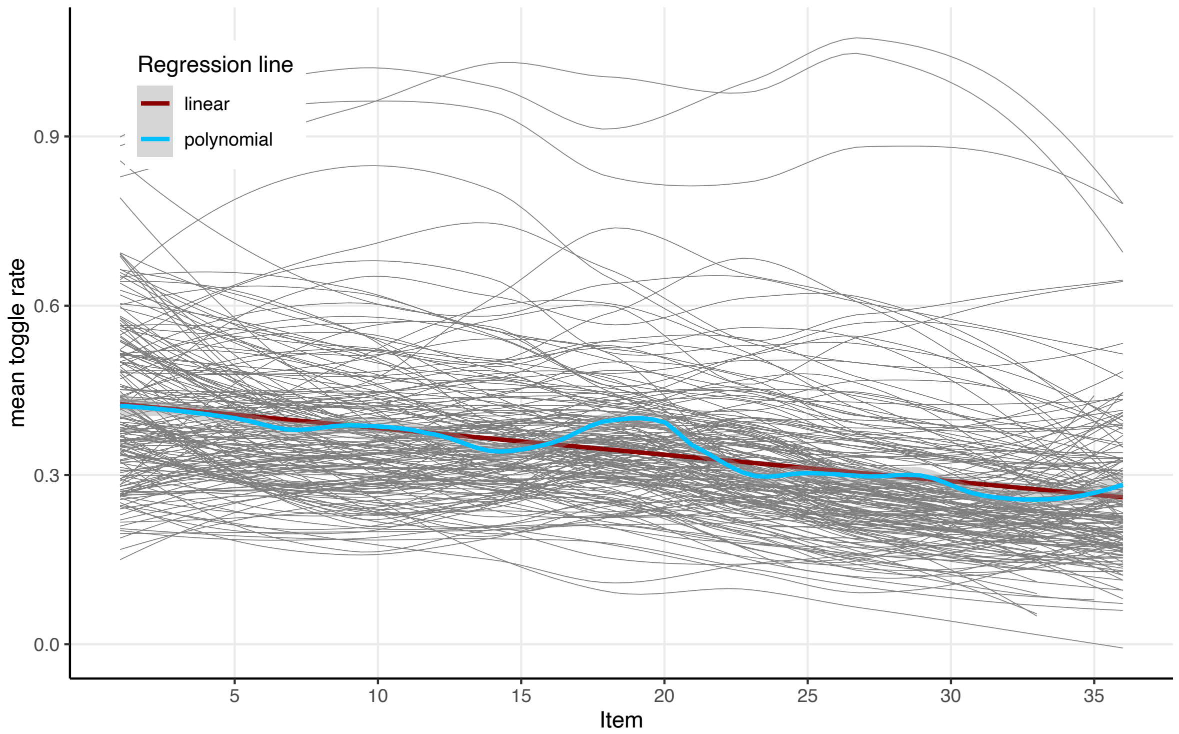

Figure S6_33. Toggle rate for each participant (gray), linear (red) and local polynomial (blue) regression fitted line for analysed sample. Values are based on all 36 items of participants who completed at least the first 33 items.

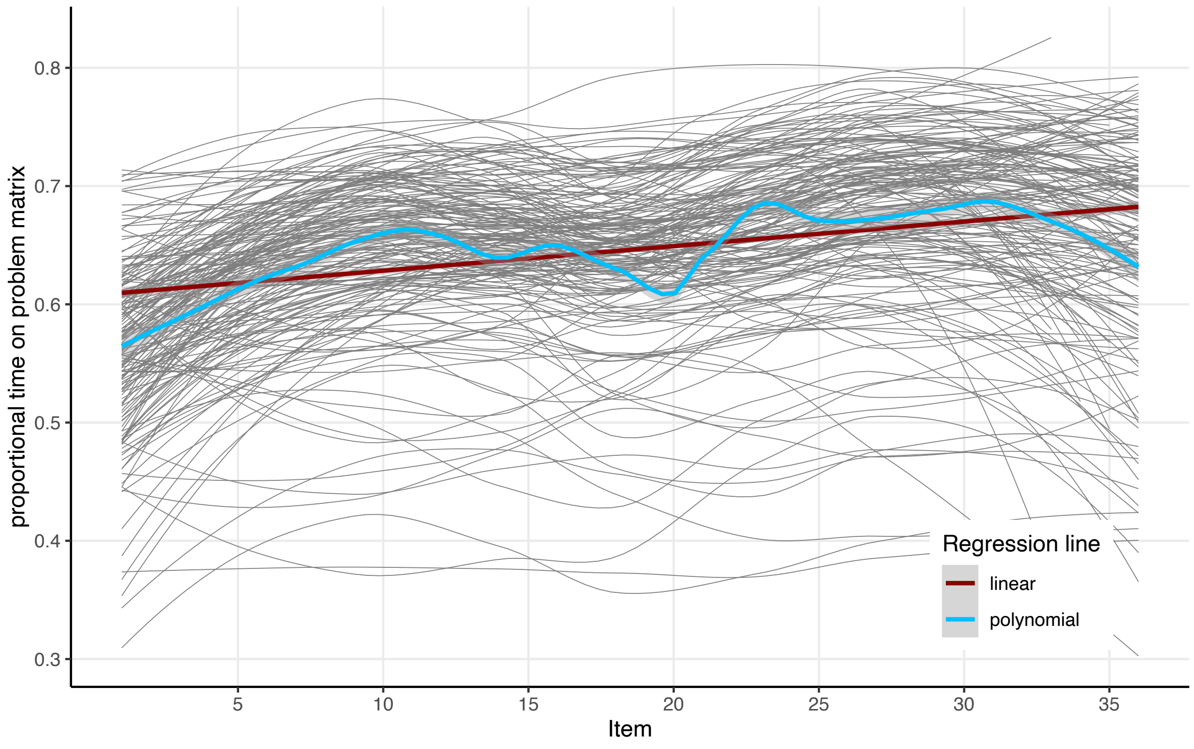

Figure S7_33. Proportional time on problem matrix for each participant (gray), linear (red) and local polynomial (blue) regression fitted line for analysed sample. Values are based on all 36 items of participants who completed at least the first 33 items.

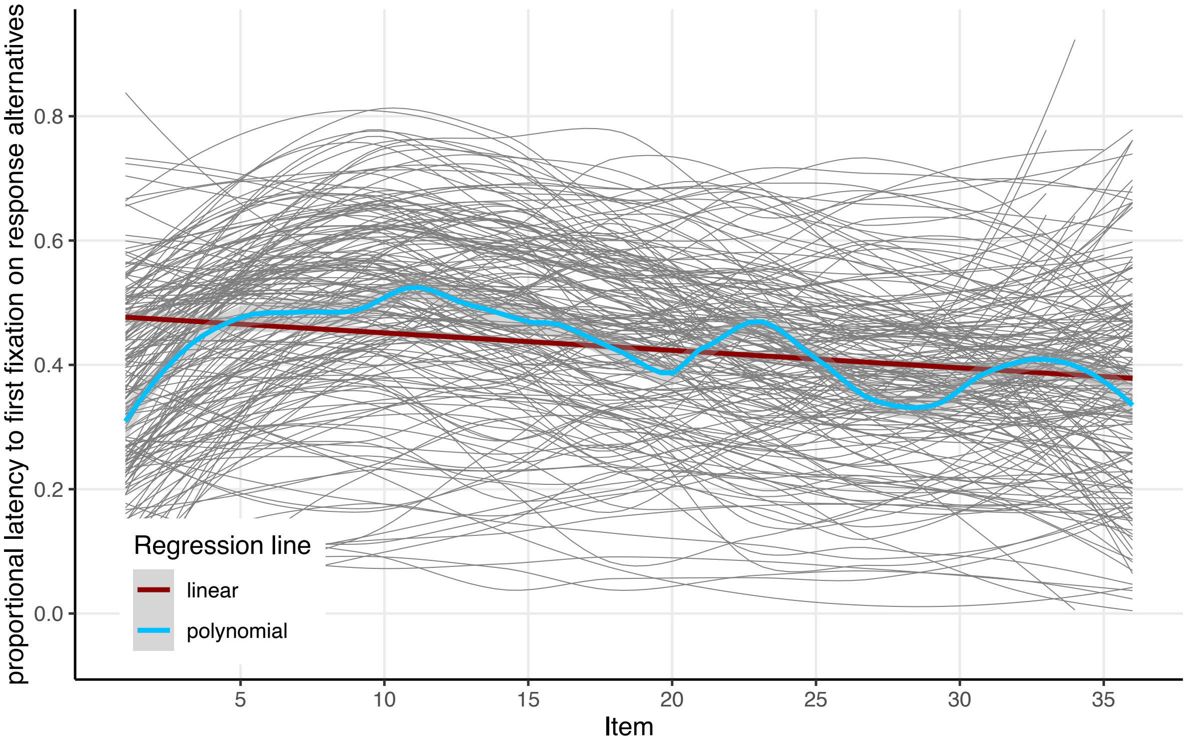

Figure S8_33. Proportional time to first fixation on response alternatives for each participant (gray), linear (red) and local polynomial (blue) regression fitted line for analysed sample. Values are based on all 36 items of participants who completed at least the first 33 items.

Table S1_33. Correlation matrix of APM score, eye tracking metrics and strategy questionnaire for participants completing 33 items on the APM.

	APM	Tog Rate	N of Tog	I-Latency	T on M	T on RA	FF on RA	Pr. T on M	Pr. T on RA	Pr. T to FF
APM - Score	APM	0.000	0.006	0.000	0.000	0.427	0.000	0.000	0.000	0.000
Toggle Rate	-0.628	Tog Rate	0.000	0.000	0.000	0.649	0.000	0.000	0.000	0.000
Number of Toggles	-0.200	0.438	N of Tog	0.000	0.000	0.000	0.001	0.004	0.000	0.000
Item Latency	0.492	-0.515	0.423	I-Latency	0.000	0.000	0.000	0.000	0.000	0.576
Time on Matrix	0.574	-0.585	0.297	0.966	T on M	0.000	0.000	0.000	0.000	0.242
Time on Response Alternatives	*0.059*	*0.034*	0.746	0.630	0.526	T on RA	0.397	0.873	0.000	0.000
Latency to First Fixation on RA	0.581	-0.750	-0.250	0.608	0.673	*0.063*	FF on RA	0.000	0.000	0.000
Proportional Time on Matrix	0.565	-0.671	-0.210	0.414	0.605	*-0.012*	0.564	Pr. T on M	0.000	0.000
Proportional Time on Response Alternatives	-0.481	0.769	0.301	-0.432	-0.495	0.321	-0.621	-0.598	Pr. T on RA	0.000
Proportional Time to First Fixation on RA	0.488	-0.660	-0.724	*-0.041*	*0.086*	-0.478	0.688	0.490	-0.551	Pr. T to FF

Note. Given are correlations in the lower triangular matrix (non- significant values are *italic*), with the corresponding *p*-value in the upper triangular matrix. Abbreviations in the first row and diagonally across the matrix correspond to information given in the first column. APM Score was calculated as the number of correct answers given within the first 33 items. Values are based on all 33 items of participants who completed at least the first 33 items.

Table S2_33. Goodness-of-fit indices and criteria for each of the calculated measurement models.

	χ^2 (*df*)	*p*	CFI	RMSEA	SRMR	AIC
APM scores						
one-factor model / Model A	610.04 (495)	<.001	0.898	0.036	0.061	6487
**bifactor model / Model B**	**567.41 (494)**	0.012	0.935	0.029	0.060	6446
bifactor model / Model C	572.74 (494)	0.008	0.930	0.030	0.060	6451
Toggle rate						
one-factor model / Model A	696.74 (495)	<.001	0.901	0.050	0.047	-6383
bifactor model / Model B	650.09 (494)	<.001	0.923	0.044	0.047	-6426
**bifactor model / Model C**	**650.88 (494)**	**<.001**	**0.922**	**0.044**	**0.045**	**-6427**
Proportional Time on Matrix						
one-factor model / Model A	718.61 (495)	<.001	0.913	0.052	0.050	-13752
bifactor model / Model B	660.93 (494)	<.001	0.936	0.045	0.053	-13823
**bifactor model / Model C**	**651.55(494)**	**<.001**	**0.940**	**0.043**	**0.048**	**-13835**
Latency to first fixation on response alternatives						
one-factor model / Model A	643.15 (495)	<.001	0.889	0.042	0.064	-599
**bifactor model / Model B**	573.67 (494)	0.008	0.940	0.031	0.062	-669
bifactor model / Model C	580.43 (494)	0.004	0.935	0.032	0.060	-662
Full Models with Model B for the APM score data and the best fitting model for the respective eye tracking metric						
Toggle rate / Model C	2549.17 (2073)	<.001	0.859	0.036	0..061	-88
Proportional time on Matrix / Model C	2878.08 (2073)	<.001	0.834	0.044	0.066	-7461
Proportional time to first fixation on response alternatives / Model B	2692.37 (2145)	<.001	0.796	0.041	0.068	5640

Note. Model A is the one-factor model. Model B includes a second latent variable with linearly, and Model C with quadratically increasing factor loadings from the first to the last item. Models in **bold** indicate the best fitting model.

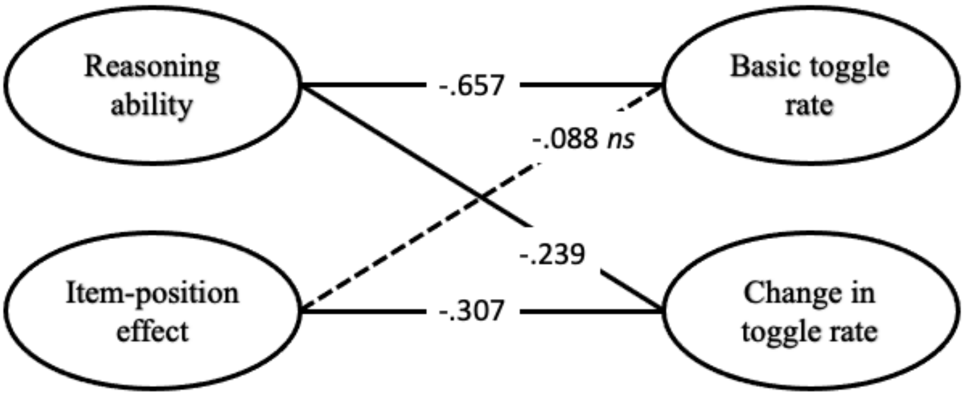

Figure S9_33. Correlations between latent variables of the final model regarding toggle rate.

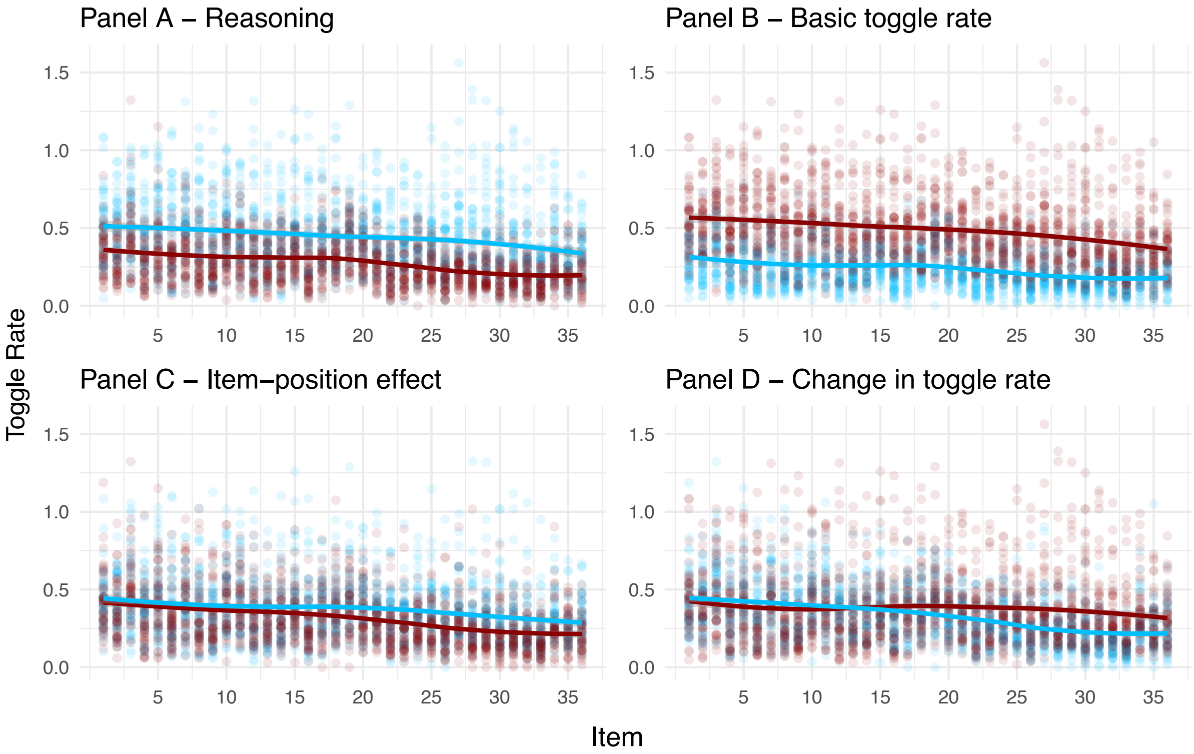

Figure S10_33. Toggle rate for participants with high or low values on the latent variables. *Note.* Blue lines represent participants with low factor scores on the latent variable. Red lines represent participants with high factor scores on the depicted latent variable.

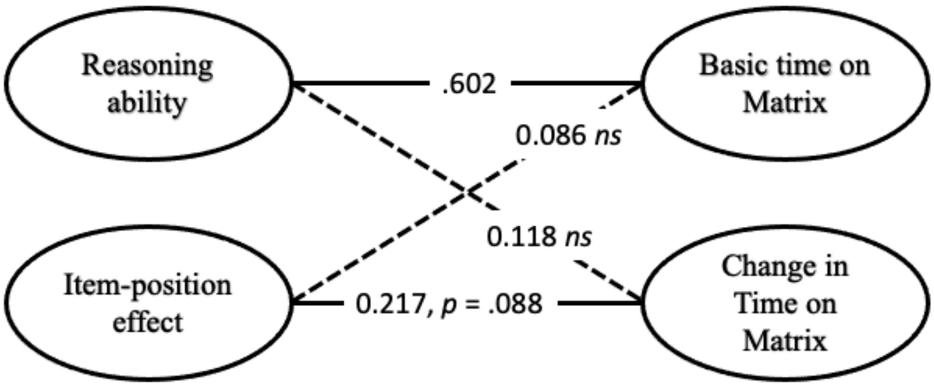

Figure S11_33. Correlations between latent variables of the final model regarding the proportional time on the problem matrix.

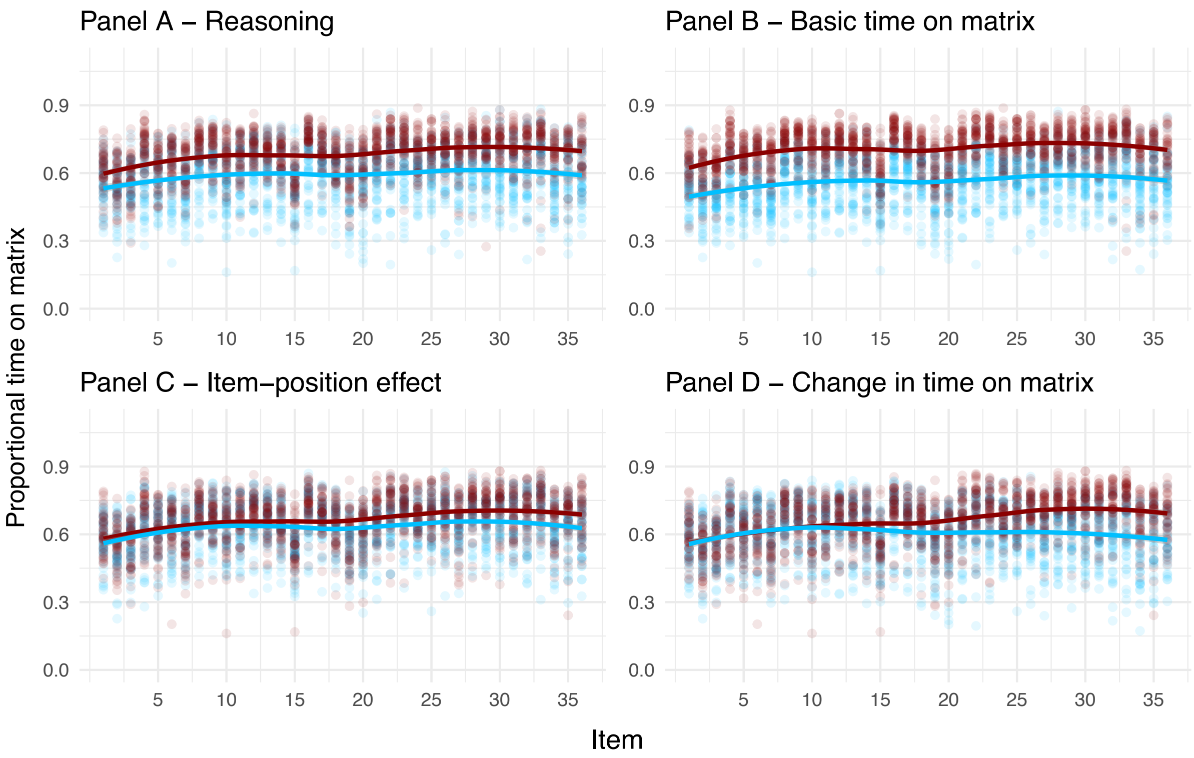

Figure S12_33. Proportional time on matrix for participants with high or low values on the latent variables. *Note.* Blue lines represent participants with low factor scores on the latent variable. Red lines represent participants with high factor scores on the depicted latent variable.

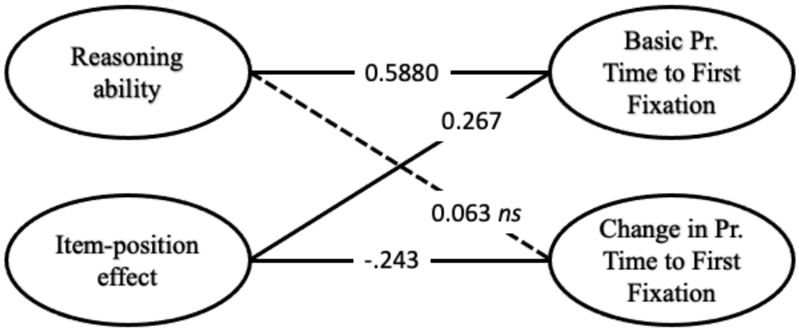

Figure S13_33. Correlations between latent variables of the final model regarding the proportional time to first fixation on response alternatives.

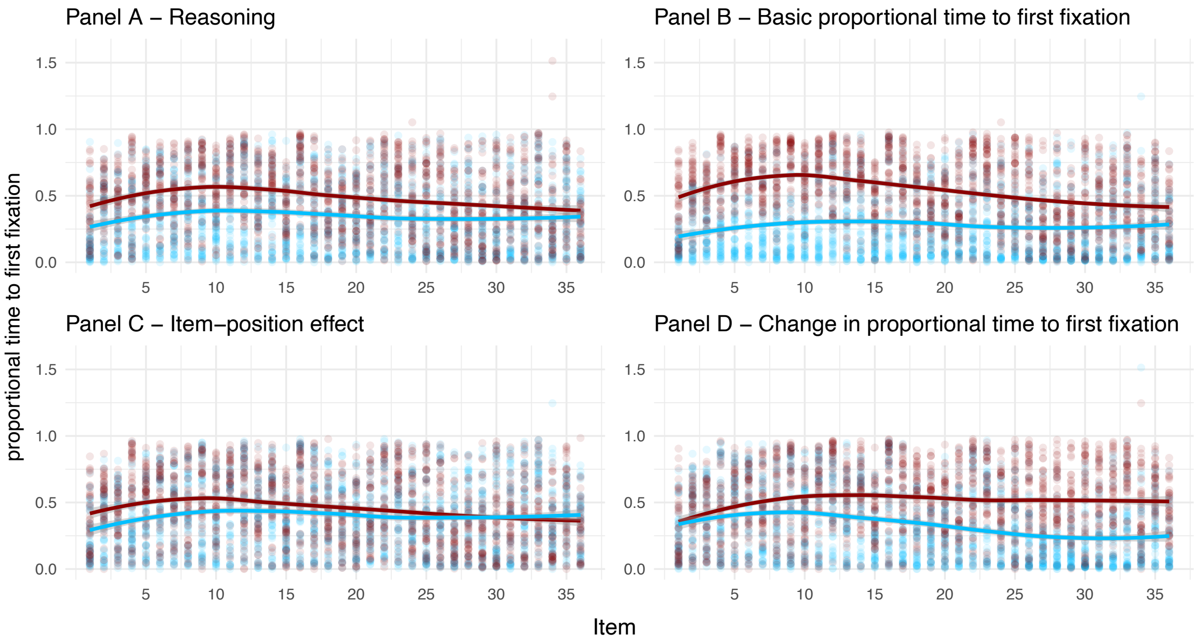

Figure S14_33. Proportional time to first fixation on response alternatives for participants with high or low values on the latent variables. *Note.* Blue lines represent participants with low factor scores on the latent variable. Red lines represent participants with high factor scores on the depicted latent variable.

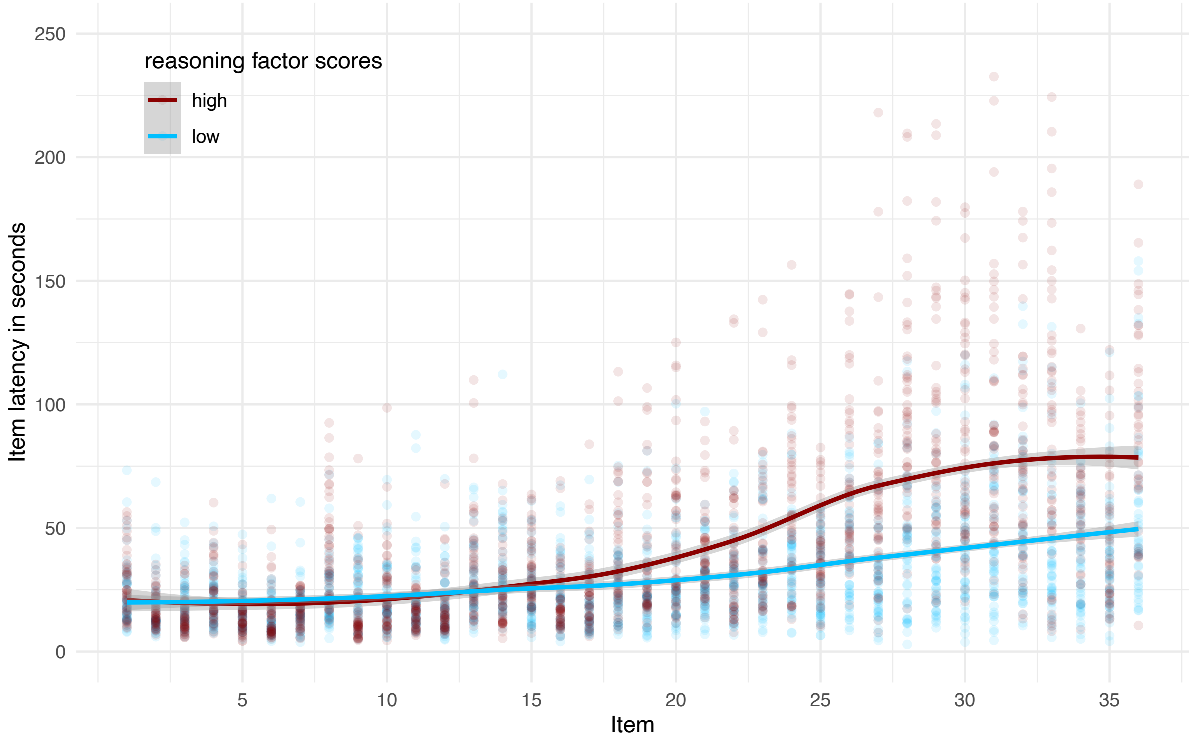

Figure S15_33. Item Latencies for participants with high or low reasoning ability factor scores. Note. Given are item latencies in seconds and a polynomial trend line for the participants with the 50 highest (in red) and 50 lowest (in blue) reasoning ability factor scores of the final model.
